# What determines if a ligand activates or passivates a superatom cluster?†
†Electronic supplementary information (ESI) available: Details of both experimental and theoretical (Fig. S1–S37), energies and reaction coordinates for the Al_*n*_I_*m*_^–^ clusters, as well as coordinates for all these clusters. See DOI: 10.1039/c5sc04293c


**DOI:** 10.1039/c5sc04293c

**Published:** 2016-01-27

**Authors:** Zhixun Luo, Arthur C. Reber, Meiye Jia, William H. Blades, Shiv N. Khanna, A. W. Castleman

**Affiliations:** a State Key Laboratory for Structural Chemistry of Unstable and Stable Species , Institute of Chemistry , Chinese Academy of Sciences , Beijing , 100190 , China . Email: zxluo@iccas.ac.cn; b Departments of Chemistry and Physics , The Pennsylvania State University , University Park , PA 16802 , USA . Email: awc@psu.edu; c Department of Physics , Virginia Commonwealth University , Richmond , VA 23284 , USA . Email: snkhanna@vcu.edu

## Abstract

Quantum confinement in small metal clusters leads to a bunching of states into electronic shells reminiscent of shells in atoms, enabling the classification of clusters as superatoms. The addition of ligands tunes the valence electron count of metal clusters and appears to serve as protecting groups preventing the etching of the metallic cores. Through a joint experimental and theoretical study of the reactivity of methanol with aluminum clusters ligated with iodine, we find that ligands enhance the stability of some clusters, however in some cases the electronegative ligand may perturb the charge density of the metallic core generating active sites that can lead to the etching of the cluster. The reactivity is driven by Lewis acid and Lewis base active sites that form through the selective positioning of the iodine and the structure of the aluminum core. This study enriches the general knowledge on clusters including offering insight into the stability of ligand protected clusters synthesized *via* wet chemistry.

## Abstract 2

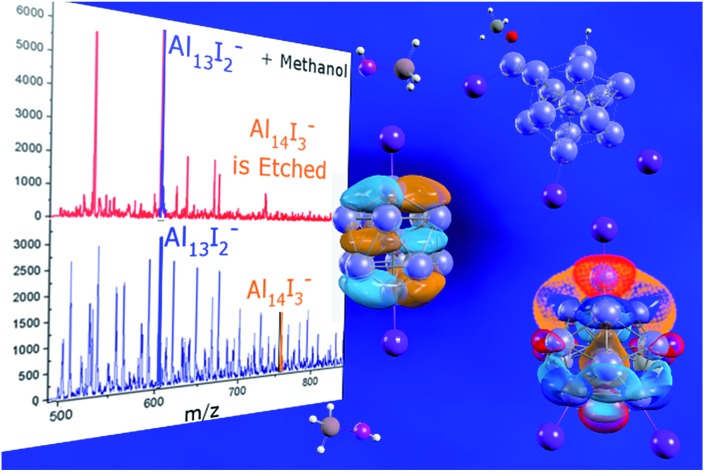
Quantum confinement in small metal clusters leads to a bunching of states into electronic shells reminiscent of shells in atoms, enabling the classification of clusters as superatoms.

## Introduction

Considerable research over the past three decades has shown that small clusters containing a few to a few hundred atoms exhibit novel properties that change non-monotonically with size and shape, offering the prospect for better functional materials.^1^,^2^ Studies in cluster physics and cluster chemistry also provide information on the evolution in behaviour from the atomic scale to the solid state. The quantum states in small compact metal clusters group into shells that can be described within the confined Nearly Free Electron Gas (NFEG) model.^3^–^9^ The electronic states correspond to delocalized orbitals 1S^2^, 1P^6^, 1D^10^, 2S^2^… and when the valence electron count refers to a filled electronic shell, the cluster exhibits enhanced stability and reduced reactivity. The grouping of electronic states into shells and the associated stability and reactive patterns has enabled the description of clusters with well-defined valences as superatoms forming a new dimension to the periodic table of elements.

In the gas phase, electronic shells explain cluster reactivity with oxygen, where magic clusters with electron counts corresponding to closed electronic shells like Al_13_^–^ and Al_23_^–^*etc.* were found to be resistant towards oxygen etching.^10^–^14^ For example, the ground state of Al_13_^–^ with 40 valence electrons corresponds to the filled shells of |1S^2^|1P^6^|1D^10^2S^2^|1F^14^2P^6^| with a HOMO–LUMO gap of 1.83 eV to the 2D^10^ unfilled shell. The decrease in reactivity of these clusters with O_2_ is largely associated with the triplet ground state of O_2_ where the lowest unfilled orbitals are minority spin pi* orbitals. Any activation of the O_2_ molecule requires the filling of these minority pi* orbitals which entails a spin excitation of the cluster to conserve the overall spin of the reactive complex. This activation energy is determined by the HOMO–LUMO gap and this explains why clusters with filled electronic shells and large HOMO–LUMO gaps are found to be unreactive towards O_2_.^15^,^16^ Through studies on the oxygen etching of Al_*n*_^–^,^15^ Mg_*n*_Al_*m*_^–^,^13^ CuAl_*n*_^–^,^17^ Al_*n*_H_*m*_^–^,^18^ Al_*n*_C^–^,^19^ and Al_*n*_B^–^ clusters,^20^ it has been found that clusters with HOMO–LUMO gaps exceeding 1.2 eV are all resistant to oxygen etching in flow tube experiments. Therefore, oxygen etching serves as a means to probe the filled electronic shells and the superatomic character of clusters.^1^,^21^ Since the valence electron count can be controlled by adding halogens, further studies on reactivity of Al_*n*_I_*m*_^–^ clusters with oxygen showed that Al_13_I_2*n*_^–^ and Al_14_I_2*n*+1_^–^ (*n* = integer) clusters were resilient to reactivity with oxygen.^22^,^23^ These results allowed the classification of Al_13_ as a halogen superatom and Al_14_ as an alkaline earth superatom.

Our studies on the reactivity of Al_*n*_^–^ clusters with water, methanol and formaldehyde indicated that, unlike the case of atoms where a large HOMO–LUMO gap could lead to chemical inertness, the reactivity of superatoms with such polar molecules had a different fundamental origin.^24^–^32^ Species that have a non-uniform distribution of charge density are marked by Lewis acid sites that accept charge and Lewis base sites that donate charge. The Lewis acidity, rather than the charge acceptance, is most important because in the reaction with water and alcohols, the lone pair of the oxygen inserts into the aluminium cluster, so the cluster must accept an electron pair. The clusters with these complementary Lewis acid/base pairs are highly reactive with protic species. Thus, the chemical stability of a small metal cluster is maximized when, (i) the cluster has a closed electronic shell that corresponds to a HOMO–LUMO gap larger than 1.2 eV; and (ii) the charge density is evenly distributed over the surface of the cluster preventing the presence of active sites. These two criteria are also connected to other properties that correlate with reactivity such as higher ionization potentials, detachment energy and, as we show, reaction barriers.

Ligand protected metal clusters synthesized *via* wet chemistry have also generated extensive interest, and the stability of these ligated clusters are also explained using the superatom concept.^33^–^38^ Ligands such as thiols, phosphines and halides surrounding the noble metallic core are used to alter the electronic structure of the metal cluster leading to a delocalized electron count that corresponds to a filled electronic shell. These ligands are also used as passivating and protecting groups.^35^,^37^,^39^–^43^ For example, several aluminium cluster assembled materials have been synthesized including those based on Al_77_ and icosahedral Al_12_ motifs.^44^–^46^ Al_77_ is particularly interesting as the cluster has a highly spherical shape and an odd number of electrons and does not have a closed electronic shell.^46^ Numerous ligand protected clusters have been synthesized by Schnöckel and co-workers revealing the rich chemistry of metalloids.^47^,^48^ Irrespective of whether the cluster may be characterized as a metal or metalloid cluster, the fact that these clusters form materials implies that electronic shell closure is not the only criteria for material assembly. Also, as we have shown, the selective positioning of the ligands can be used to distort the charge density over the surface of the aluminium cluster resulting in active sites.^28^ The addition of a ligand can therefore protect a cluster enhancing stability or make it more reactive. The question is under what circumstances do ligands passivate or activate a superatom cluster?

We have identified two series of stable aluminium iodide clusters Al_13_I_*n*_^–^ (*n* = 0, 2, 4) and Al_14_I_*m*_^–^ (*m* = 3, 5) by observing their stability in oxygen etching experiments.^23^ Here we clarify how ligands can be used to control the reactivity of metal clusters. We have performed a synergistic experimental and theoretical study of the etching of Al_*n*_I_*m*_^–^ clusters with methanol. Due to its clean etching spectrum, and because its vapour pressure is higher than water, methanol was chosen as the etchant. The cluster reaction experiments were conducted in a fast flow reactor, and theoretical studies of the reactivity and active sites of Al_*n*_I_*m*_^–^ were emphasized for *n* = 7–14 and *m* = 0–2. We find that Al_14_I_3_^–^ is reactive due to its activated adatom structure, where an aluminium atom with iodine lies on top of the icosahedral core. This activated adatom is found to serve as a Lewis acid site. In contrast, the Al_13_I_*m*_^–^ clusters (*m* = 0–3) and Al_7_I_2_^–^ are unreactive with methanol because of their symmetric core and their even distribution of charges around the surface of the cluster. Our findings not only explain the stability and reactivity of aluminium iodide clusters, but also provide new insight into the fundamental mechanism that prevent etching and the origin of stability in ligated-metal clusters.

## Materials and methods

### Cluster production and reactivity

The clusters were generated using a laser vaporization (LaVa) source,^13^ made of stainless steel, with an external motor to control the target rotation of Al rod (Kurt J. Lesker, 99.999% purity, *Φ* 6 mm), as well as a gas inlet connection to a constant flow of helium (Praxair, Inc., purity > 99.995%). Solid iodine (Sigma-Aldrich, 99.999%) was added in the LaVa-source container to form aluminium iodide clusters. The outlet expansion nozzle was made of a Teflon tube (∼2.5 cm length) with an inner diameter of 0.32 cm. The pressure inside the source during operating conditions was kept at ∼20 Torr, suggesting a Knudsen number of ∼2.8 × 10^–3^ and a terminal Mach number of ∼12.3. The helium buffer gas introduced from the inlet of the source carried the clusters through the nozzle into a flow tube where they encountered and reacted with methanol (Sigma-Aldrich, >99.9%) at room temperature. The reactant methanol gas was introduced in the cluster beam ∼30 cm downstream from the source (resulting in thermalized Al_*n*_I_*m*_^–^ clusters) and allowed to react with the clusters over a 60 cm distance and time of ∼8 ms. The reaction products were extracted into a differentially pumped ion guide vacuum system, and analysed *via* a quadrupole mass spectrometer (Extrel QMS). The pressure in the reaction flow-tube was kept at ∼0.7 Torr, *i.e.*, several hundred collisions for the related reactions.

### Computational methods

Energy calculations of the ground state geometry, electronic structure, and transition state were carried out using a first principles density functional approach. The calculations used a linear combination of Slater type orbitals located at atomic sites using the Amsterdam density functional set of codes.^49^ They were carried out using the TZ2P basis within the PBE gradient corrected density functional formalism.^50^ The zeroth order regular approximation was used for relativistic effects. All structures were fully optimized without constraints. The transition states were calculated using the linear transit approach, by fixing the O–H bond distance over a range of distances and identifying the saddle point.

## Results and discussion

**Fig. 1:**
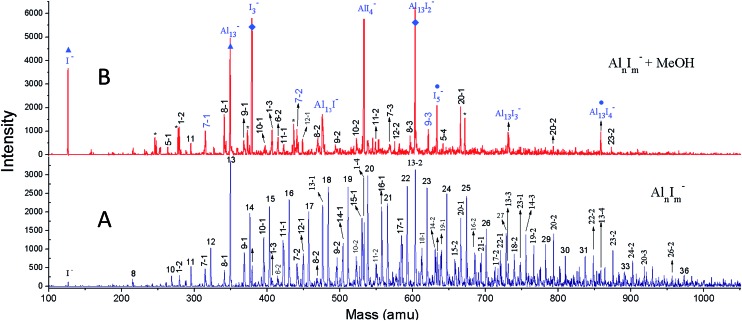
The reactivity of Al_*n*_I_*m*_^–^ clusters with MeOH. (A) The nascent mass spectrum of Al_*n*_I_*m*_^–^ clusters. (B) The spectrum of Al_*n*_I_*m*_^–^ clusters after methanol etching. A few peaks marked with * refer to Al_*n*_(CH_3_OH)_*m*_^–^; while the peaks marked with ▲,◆,[black circle] display the I^–^, Al_13_^–^; I_3_^–^, Al_13_I_2_^–^; I_5_^–^, and Al_13_I_4_^–^ species respectively.

We have achieved a well-resolved mass spectrum of Al_*n*_I_*m*_^–^ (7 < *n* < 37, *m* = 0–3) clusters by adding solid iodine into the LaVa source (∼20 Torr pressures), as shown in Fig. 1A. The Al_*n*_I_*m*_^–^ clusters display a normal distribution centred at Al_21_^–^, except for Al_13_^–^ which exhibits slightly enhanced stability. Aluminium readily reacts with iodine at room temperature and the formed aluminium iodides mainly cover Al_*n*_I_1–3_^–^, with Al_13_I^–^ and Al_13_I_2_^–^ as the most abundant. After the addition of methanol, the Al_*n*_I_*m*_^–^ abundancies undergo a dramatic change due to etching, as shown in Fig. 1B (for more details see ESI, Fig. S1 and S2†). Peaks corresponding to I^–^, I_3_^–^, AlI_4_^–^, and I_5_^–^ appear with enhanced intensities owing to the production of I^–^ in the etching reactions. Most of the Al_*n*_I_*m*_^–^ clusters with sizes larger than *n* = 13 disappear or display weakened intensities. We note that Al_13_^–^, Al_13_I_2_^–^ and Al_13_I_4_^–^ (as marked with ▲, ◆, [black circle] in Fig. 1) display analogous stability to I^–^ and hypervalent I_3_^–^/I_5_^–^ respectively surviving the methanol etching reactions (Al_13_^–^ ↔ X^–^; Al_13_I_2_^–^ ↔ X_3_^–^; Al_13_I_4_^–^ ↔ X_5_^–^).

**Fig. 2:**
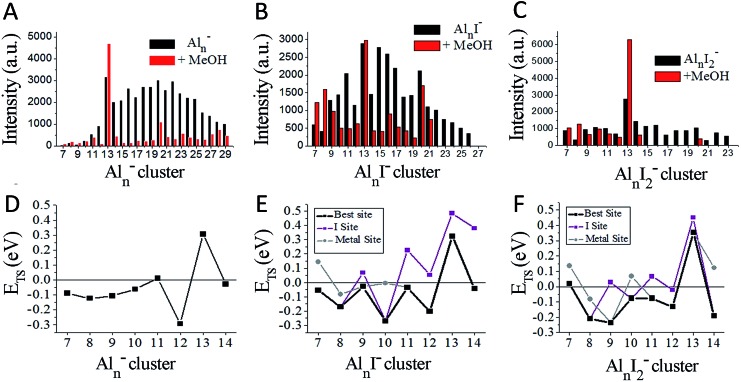
Ionic intensities and transition state energies (*E*_TS_). (A–C) The experimental ionic intensities of Al_*n*_^–^, Al_*n*_I^–^, and Al_*n*_I_2_^–^ at the absence and presence of methanol, where the intensity values correspond to the integral areas of the correlated peaks of Fig. 1. (D–F) The calculated *E*_TS_ for the cleavage of the O–H bond of methanol for Al_*n*_^–^, Al_*n*_I^–^, and Al_*n*_I_2_^–^. The structures and energetics are shown in Fig. S10–S37.† a.u. = arbitrary units.

Fig. 2A–C plot the intensities of Al_*n*_I_0–2_^–^ clusters in the absence and presence of methanol. The intensities of Al_7_I^–^, Al_8_I^–^, Al_8_I_2_^–^, Al_13_^–^, Al_13_I_2_^–^ and Al_13_I_4_^–^ are strengthened up to twice of their nascent peaks. Al_7_I_2_^–^, Al_20_I^–^, Al_13_I^–^, and Al_13_I_3_^–^ (Fig. S3†) display similar intensities before and after methanol etching. Al_13_I^–^ and Al_13_I_3_^–^ have an odd number of electrons, confirming that a closed electronic shell is not necessary for a cluster to be resistant to methanol etching. Al_14_I_3_^–^ was previously found to be resistant to oxygen etching,^23^ however the abundant Al_14_I_3_^–^ is almost completely depleted after methanol is introduced to the flow tube. Seen from Fig. 2A–C, Al_7_I^–^ and Al_7_I_2_^–^ have higher abundances after methanol etching, showing that the addition of iodine may sometimes activate a cluster.

To understand the microscopic mechanism by which these clusters react with methanol, we have investigated the lowest energy structures for Al_*n*_I_0–2_^–^ (*n* = 7–14), and also Al_9_I_3_^–^ and Al_14_I_3_^–^. Fig. S4 and S5† show the structures, and Fig. S6† shows the HOMO–LUMO gaps, aluminium and iodine binding energies. Several clusters have HOMO–LUMO gaps that are large enough that the cluster may be considered to have a closed electronic shell. For example, Al_7_I_2_^–^ has a gap of 1.73 eV; Al_13_^–^ and Al_13_I_2_^–^ have gaps of 1.81 eV and 1.59 eV respectively, and Al_14_I_3_^–^ has a gap of 1.35 eV. Previous studies have indicated that clusters with HOMO–LUMO gaps higher than 1.2 eV are generally non-reactive towards oxygen.^13^–^15^ The electronic structure of Al_13_^–^, Al_14_I_3_^–^, and Al_7_I_2_^–^ are shown in Fig. S7† for reference. As pointed out in the introduction, these clusters are expected to be non-reactive with oxygen.

To understand the reactivity with methanol, we have calculated the lowest energy transition state for the breaking of the O–H bond on the clusters surface of Al_*n*_^–^, Al_*n*_I^–^ and Al_*n*_I_2_^–^ respectively. In our previous studies on Al_*n*_^–^ clusters, we found that water and methanol only form complexes when the O–H bond is broken.^21^–^23^ Fig. 2D displays the transition state energies, *E*_TS_, for the Al_*n*_^–^ clusters. We have used the *E*_TS_, as shown in eqn (1), as a measure of the reactivity in the gas phase. The reaction may proceed rapidly when the *E*_TS_ is negative, and the reaction will proceed slowly when the *E*_TS_ is positive.

1 *E*_TS_ = *E*(Al_*n*_I_*m*_(CH_3_O–H)_TS_^–^) – *E*(CH_3_OH) – *E*(Al_*n*_I_*m*_^–^)

The *E*_TS_ is most applicable in gas phase reactions, because the energy gained by the adsorption of the reactant remains in the cluster. In solution, the activation energy, the energy difference between the methanol–cluster complex and the transition state, will be more important. This is due to the fact that the energy gained by complex formation is more rapidly dissipated into the surrounding environment. Another way to consider this is, if the energy required to cleave the O–H bond is lower than the energy required for desorption of the molecule, then we expect O–H cleavage to be a likely product, and when desorption is the lower energy pathway, then O–H cleavage is unlikely.

We have plotted the lowest energy transition states for each cluster under two separate circumstances: when the oxygen of the methanol attaches to an unligated aluminium–aluminium site, and when the oxygen of the methanol attaches to a site corresponding to an aluminium atom that is bound to iodine. We find that Al_11_^–^ and Al_13_^–^ have positive *E*_TS_ values, which is consistent with the experimental finding that Al_13_^–^ increases in intensity and Al_11_^–^ shows resistance to reaction. The remaining clusters *n* = 7–14 all show minimal abundance after methanol etching. For the Al_*n*_I^–^ series in Fig. 2B and E, we find only Al_13_I^–^ to have a positive *E*_TS_, and Al_13_I^–^ is experimentally the most abundant species in the series. The binding energies of methanol are shown in Fig. S8.† The experiment also finds Al_7_I^–^ and Al_8_I^–^ to have increased abundance, while theory finds them to be reactive indicating that these clusters are likely products of fragmentation of larger clusters. For the Al_*n*_I_2_^–^ series in Fig. 2C and F, theory finds that Al_7_I_2_^–^, and Al_13_I_2_^–^ have positive *E*_TS_, which is consistent with the experimental results by noting that Al_13_I_2_^–^ is by far the most abundant cluster in this series while Al_7_I_2_^–^ is the third most abundant.

**Fig. 3:**
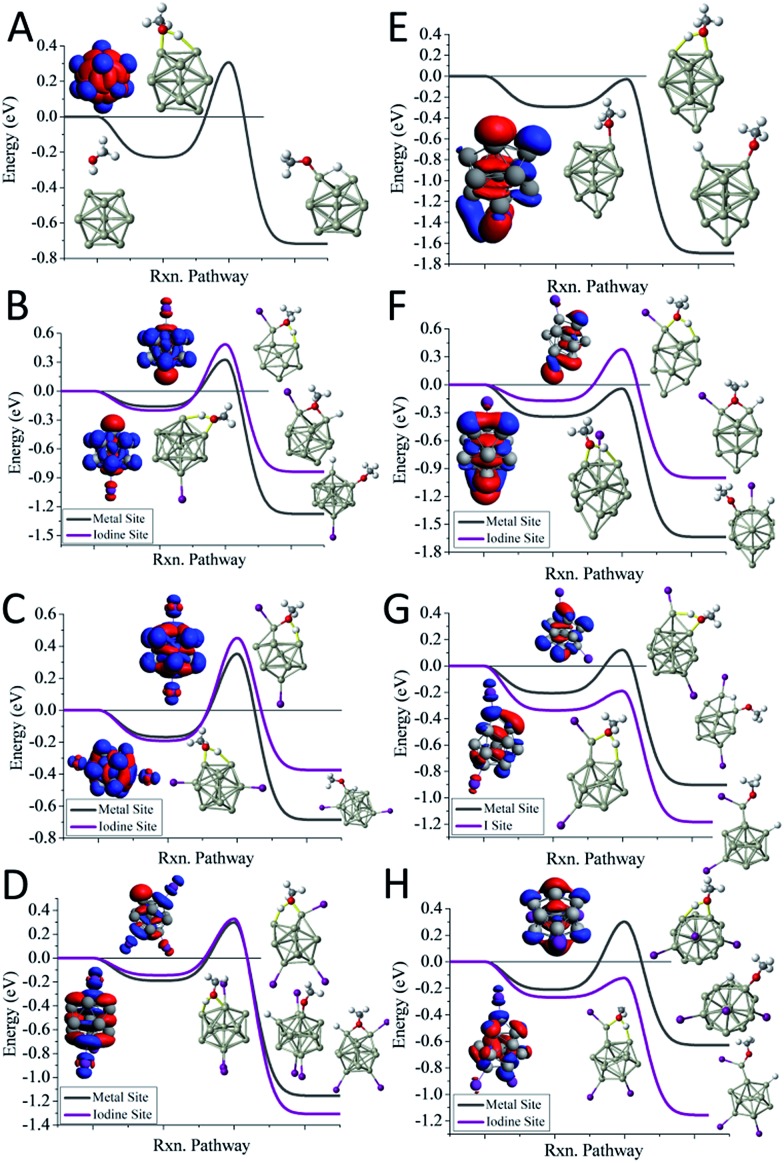
The reaction coordinates for Al_13_I_0–3_^–^ and Al_14_I_0–3_^–^. The reaction pathways with MeOH for (A) Al_13_^–^, (B) Al_13_I^–^, (C) Al_13_I_2_^–^, (D) Al_13_I_3_^–^, (E) Al_14_^–^, (F) Al_14_I^–^, (G) Al_14_I_2_^–^, (H) Al_14_I_3_^–^. The red indicates the HOMO or near HOMO orbitals for degenerate orbitals, and blue indicates LUMO or LUMO+1 orbitals that are fully unoccupied.

We first examine the reactivity of the Al_13_I_*m*_^–^ superatomic clusters with methanol. Fig. 3A shows the reaction pathway for Al_13_^–^ with methanol. Al_13_^–^ has a closed electronic shell and an icosahedral geometric structure. The LUMO orbitals are plotted in blue, and the HOMO orbitals are plotted in red. The closed electronic shell demonstrates that the frontier orbitals are evenly distributed over the surface of the cluster, precluding the presence of active sites. The oxygen atom of methanol does not readily bind to the cluster, demonstrating that Al_13_^–^ is not a good Lewis acid. The non-dissociative binding energy of methanol is only 0.23 eV, and the O atom prefers not to bind to the aluminium cluster. The *E*_TS_ of Al_13_^–^ is 0.31 eV indicating that this cluster is resistant to etching. The addition of iodine results in Al_13_I^–^ having a Lewis base site on the opposite side of the cluster as the iodine ligand site, while the LUMO density is evenly distributed over the surface of the metal cluster. The *E*_TS_ of Al_13_I^–^ is 0.32 eV, indicating that Al_13_I^–^ will also be resistant to methanol etching. When we investigate the cleavage of the O–H bond at the iodine site, we find that the *E*_TS_ of Al_13_I^–^ increases to 0.49 eV. Thus, iodine fails to activate the Al_13_^–^ cluster. The reason for this is that the Lewis acidity of the Al_13_I^–^ cluster is still poor because the induced active site is half filled and cannot serve as a Lewis acid, which accepts an electron pair. This weak Lewis acidity is indicated by the weak non-dissociative binding of methanol of only 0.16 eV.

The reaction pathway of Al_13_I_2_^–^ is shown in Fig. 3C, and the *E*_TS_ is 0.35 eV. The two iodine atoms lie on opposite sides of the cluster, quenching each other's induced active sites. Al_13_I_2_^–^ remains a poor Lewis acid, with the LUMO charge density evenly distributed over the icosahedral core of the cluster, and the binding energy being only 0.17 eV. Al_13_I_3_^–^ has an *E*_TS_ of 0.30 eV, and the lowest energy transition state lies at the metal site, with O binding to the induced active site, and the H atom binding to the HOMO–1. The addition of iodine to Al_13_^–^ maintains the pure cluster's resistance to reaction with methanol because the cluster remains a poor Lewis acid due to its closed geometric shell. The high abundance of the Al_13_I^–^, and Al_13_I_2_^–^ in the experiment after exposure to methanol confirms this hypothesis.

The reaction pathway of Al_14_^–^ with methanol is shown in Fig. 3E. This cluster has a 13-atom icosahedral structure with the 14^th^ atom attached as an adatom. The adatom induces a Lewis acid/base pair on the opposite side of the adatom, and the lowest energy transition state is found at this site. The *E*_TS_ is found to be –0.03 eV, indicating that the cluster is reactive. The non-dissociated methanol binding energy is 0.30 eV, implying that the cluster's Lewis acid strength is typical for an aluminium cluster anion. The reaction coordinate for Al_14_I^–^ with methanol is shown in Fig. 3F. The Al_14_ core of Al_14_I^–^ has a similar structure to pure Al_14_^–^, and the iodine atom is bound opposite site to the adatom. The frontier orbitals reveal a Lewis acid/base complementary active site adjacent to the iodine atom on the cluster. The lowest energy transition state is located at the complementary active site, and *E*_TS_ is –0.04 eV, implying a fast reaction with methanol, and the methanol binding energy is 0.34 eV, suggesting that the addition of iodine makes the cluster a marginally better Lewis acid than Al_14_^–^. The lowest energy transition state at the icosahedral iodine site is found to be unreactive, with a transition state energy of +0.38 eV greater than the reactants. Al_14_I_2_^–^ has a metallic core with a 13 atom icosahedron and an adatom, with one iodine bound to the adatom and another to an Al atom on the opposite side of the cluster. The *E*_TS_ at the iodine site is –0.19 eV, indicating that the cluster reacts rapidly (Fig. 3G). The lowest energy transition state at the metal site is +0.12 eV, suggesting that the cluster will only react at the adatom iodine site. The electronic structure reveals that LUMO+3 orbital, that lies 0.53 eV above the LUMO, is localized on the adatom and is the orbital that serves as a Lewis acid site. Al_14_I_3_^–^ has a similar geometry as Al_14_I_2_^–^, with the third iodine atom added at a second icosahedral aluminium atom opposite the adatom. The *E*_TS_ at the iodine site is –0.12 eV, indicating that the cluster should react rapidly with methanol (Fig. 3H). The *E*_TS_ at the metal site is +0.30 eV, revealing that Al_14_I_3_^–^ will react rapidly only at the adatom site. The methanol will react rapidly at the ligand activated adatom sites of Al_14_I_2_^–^ and Al_14_I_3_^–^ while being slow to react at the metal sites, which is confirmed by the nearly complete etching of Al_14_I_2_^–^ and Al_14_I_3_^–^ in the experimental spectra.

**Fig. 4:**
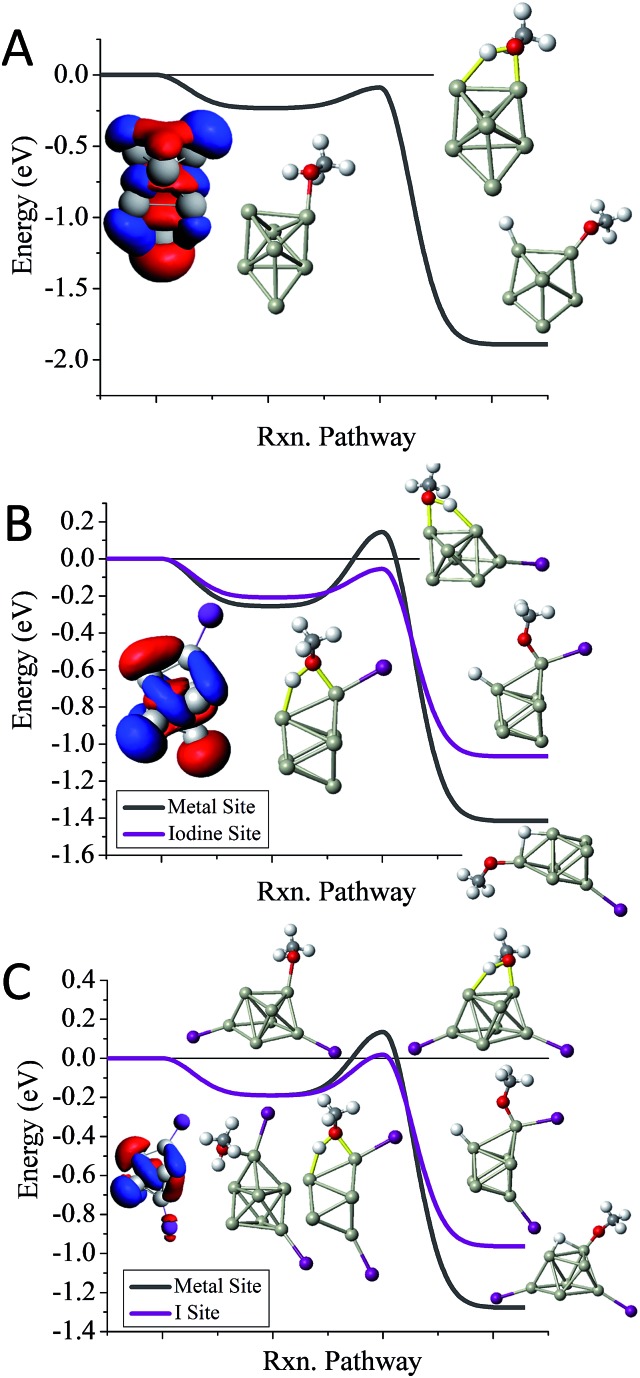
The reaction coordinates for Al_7_I_0–2_^–^ with MeOH. The calculated reaction pathways for (A) Al_7_^–^, (B) Al_7_I^–^, and (C) Al_7_I_2_^–^ with MeOH. The red indicates the HOMO or near HOMO orbitals for degenerate orbitals, and blue indicates LUMO or LUMO+1 orbitals that are fully unoccupied. Rxn pathway is abbreviated for cluster reaction coordinate and pathway.

To understand whether the addition of iodine can passivate a cluster as it gains a closed electronic shell, we investigate the Al_7_I_0–2_^–^ clusters. Al_7_^–^ reacts readily with methanol in experiments, and has an *E*_TS_ of –0.09 eV as shown in Fig. 4A. The structure of Al_7_^–^ is an octahedron with an adatom. The frontier orbitals indicate that the Al atoms on the opposite side of the adatom may serve as complementary active sites. In Al_7_I^–^ the iodine atom adds to the Al adatom site, and the resulting activated adatom has a transition state energy of –0.05 eV, indicating that the cluster should react with methanol, as shown in Fig. 4B. The metal site has a low energy transition state of +0.14 eV, suggesting that the addition of iodine has passivated the cluster's metallic core.

Al_7_I_2_^–^ is passivated with respect to methanol etching, with two iodine atoms on opposite sides of the cluster. The *E*_TS_ at the iodine site is +0.02 eV, greater than the energy of the reactants (Fig. 4C). The transition state at the most reactive metal site is +0.13 eV indicating that the metal core is also passivated. The Al_7_I_2_^–^ cluster still maintains its adatom-like geometry, however the addition of a second iodine ligand to balance out the first ligand increases the energy of the orbital localized on the adatom site, allowing this cluster to be less reactive. The relatively large abundance of Al_7_I_2_^–^, paired with the low abundance of Al_7_^–^ after methanol etching confirms our analysis.

**Fig. 5:**
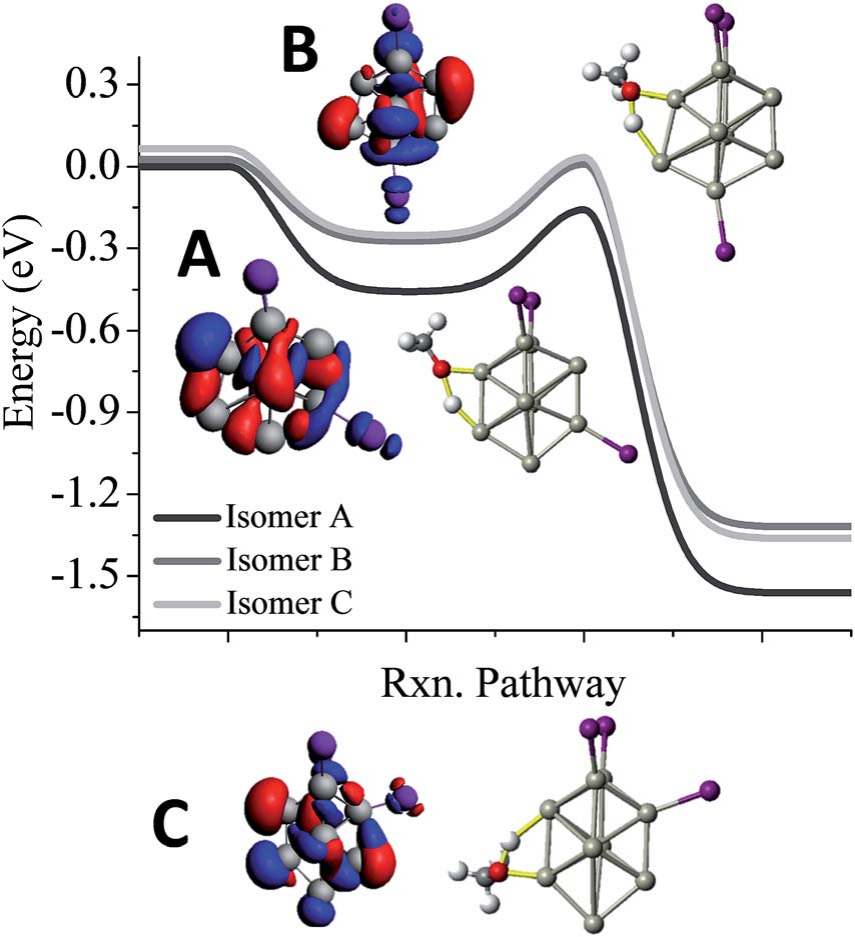
Al_9_I_3_^–^ reactivity. Reaction pathway for the three lowest energy isomers of Al_9_I_3_^–^ (A–C). The red indicates the HOMO, and the blue indicates LUMO+1. Rxn pathway is abbreviated for cluster reaction coordinate and pathway.

One of the puzzles of the experimental mass spectra is the relatively large abundance of Al_9_I_3_^–^ after methanol etching. Our investigations lead to 3 isomers, a ground state structure, and two structures with different iodine positions that are 0.03 eV and 0.06 eV higher in energy. As shown in Fig. 5, we have also investigated the reaction pathway of the three lowest energy isomers of Al_9_I_3_^–^. The lowest energy structure of Al_9_I_3_^–^, isomer A in Fig. 5, has two adjacent iodine atoms located on the octahedral core, and a third located perpendicular to the first two iodines. A Lewis acid site is found on the opposite side of the cluster as the third iodine atom, and the *E*_TS_ is –0.16 eV, showing that with unbalanced iodine atoms, the cluster is reactive. A second isomer, B in Fig. 5, has balanced ligands and lies 0.03 eV higher in energy than the ground state isomer. With the balanced ligands, the transition state for the O–H bond cleavage is only –0.02 eV, less than the energy of the reactants. This transition state is relatively high in energy making it likely to show some resistance to methanol etching. The relatively high abundance of the Al_9_I_3_^–^ cluster is due to this isomer having some resistance to methanol etching. A third isomer C possesses unbalanced ligands and has a transition state energy of –0.03 eV. This may also contribute to the observed abundance. All three isomers contain isostructural metallic cores; however, variations in the precise positioning of the iodine ligand can result in dramatic variations of cluster reactivity.

In addition, we discuss the fragmentation channels of Al_*n*_l_*m*_^–^ after methanol etching. Experimentally, we see three prominent features, (i) a decrease in the size distribution of the clusters, (ii) the abundance of I^–^, I_3_^–^, I_5_^–^, and AlI_4_^–^ all dramatically increase after the methanol etching, and (iii) there is little abundance of aluminium clusters with methoxy, Al_*n*_OCH_3_^–^. The calculated energies are shown in Fig. S9 (ESI†) in which positive energies correspond to an endothermic reaction, and negative energies correspond to an exothermic reaction. For Al_*n*_^–^, the loss of AlOCH_3_, as seen in eqn (2), is the most favourable pathway.

2 Al_*n*_^–^ + CH_3_OH → Al_*n*–1_H^–^ + AlOCH_3_

This suggests that the product after methanol etching may be HAl_*n*_^–^. For Al_*n*_I_*m*_^–^, the loss of I^–^ is a likely channel, and will produce a neutral cluster with a methoxy so it is not seen in the mass spectra.

3 Al_*n*_I_*m*_^–^ + CH_3_OH → HAl_*n*_I_*m*–1_OCH_3_ + I^–^

The I^–^ loss is endothermic for the addition of a single methanol, however, our previous studies indicated that up to 4 methanol molecules could attach to an aluminium cluster which would generate enough energy to neutralize the cluster and release I^–^.^27^ This I^–^ production is the origin of the I_3_^–^, I_5_^–^, and AlI_4_^–^ peaks that are prominent in the etching spectra.

## Conclusions

The addition of iodine ligands to aluminium superatoms may activate or passivate a cluster. To minimize the reactivity of a ligand-protected cluster, the metallic core should have a closed geometric shell with an even distribution of charges around the surface. Secondly, the ligands are most passivating when they are located in a balanced position on opposite sides of the metallic core. Al_14_I_3_^–^ is found to have a closed electronic shell, however because the metallic core has an adatom site with an iodine atom, the cluster is activated with respect to methanol. Al_13_I_2_^–^ has an icosahedral core with no adatom and balanced ligands, and is passivated to methanol. The position of the iodine may induce an active site on the opposite side of the cluster; Al_9_I_3_^–^ is reactive when the iodine atoms are unbalanced, however when the three iodine atoms are balanced on opposite sides of the cluster, the cluster is passivated. Our work demonstrates that structural features are just as important as electronic shell closure, or even more important when the synthesis is done in an oxygen-free environment. To highlight this point, Al_50_Cp_12_ is a prominent example of a cluster that is remarkably stable under an inert atmosphere and can be isolated as atomic-precise clusters. This cluster has the hallmarks of stability with a spherical metallic core and balanced ligands. However, the HOMO–LUMO gap of this cluster is only 0.85 eV, and so it is quite reactive when exposed to O_2_.^51^ This work demonstrates that even if a cluster is protected by ligands, the cluster could be reactive when the geometric considerations are not met, revealing the dual roles of electronic structure and geometric structure on the stability of bare and ligand-protected nanoparticles.

## Supplementary Material

Supplementary information
