# Supplementary material for: What determines if a ligand activates or passivates a superatom cluster?
Source: Chem Sci. 2016 Jan 27;7(5):3067–74. doi: 10.1039/c5sc04293c (PMC6005155; doi:10.1039/c5sc04293c)
Supplement: Supplementary file 1 [file SC-007-C5SC04293C-s001.pdf]

Electronic Supplementary Material (ESI) for Chemical Science.
This journal is © The Royal Society of Chemistry 2016

SUPPLEMENTARY INFORMATION

What Determines if a Ligand Activates or Passivates a Superatom Cluster?

Zhixun Luo,[†,‡,*] Arthur C. Reber,[§] Meiye Jia,[†] William H. Blades,[§] Shiv N. Khanna,[§*] and A. W. Castleman, Jr.[‡,*]

[†] State Key Laboratory for Structural Chemistry of Unstable and Stable Species, Institute of Chemistry, Chinese Academy of Sciences, Beijing, 100190, China.
[‡] Departments of Chemistry and Physics, The Pennsylvania State University, University Park, PA 16802, USA
[§] Department of Physics, Virginia Commonwealth University, Richmond, VA 23284, USA
*Correspondence to: snkhanna@vcu.edu, awc@psu.edu, zxluo@iccas.ac.cn.

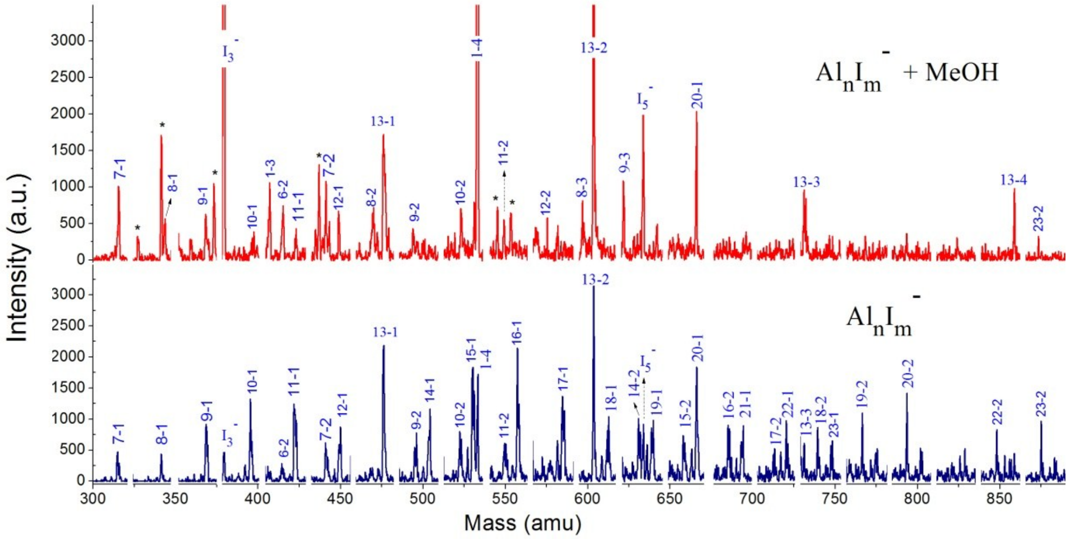

Figure S1. Methanol-etched $Al_nI_m^-$ distribution. Mass spectra of the $Al_nI_m^-$ clusters in the presence and absence of MeOH, where the peaks corresponding to pure aluminum clusters were removed to more clearly show the behavior of $Al_nI_m^-$.

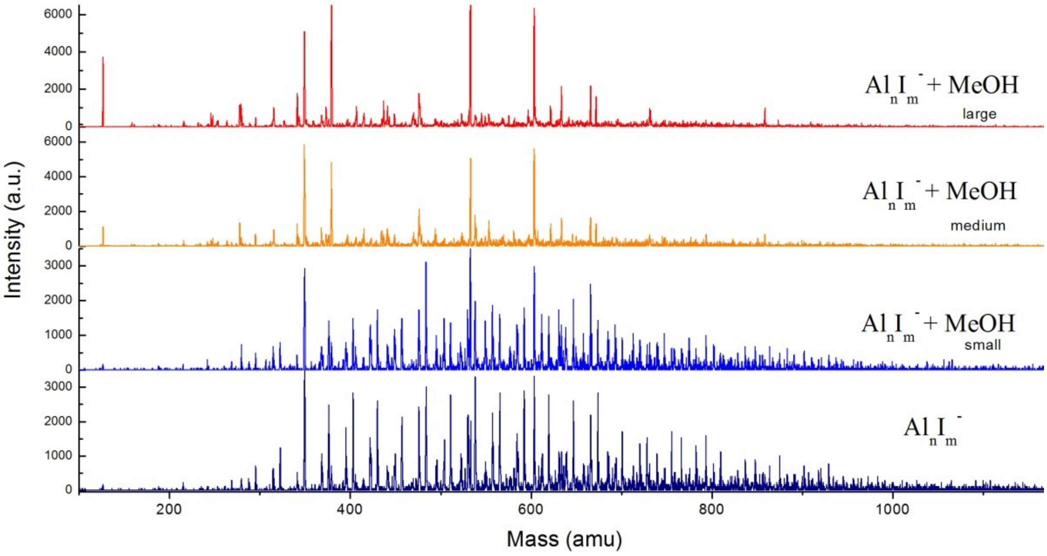

Figure S2. Flow-rate dependence. Methanol-etched $Al_nI_m^-$ distribution at different flow-rate of the methanol (~0.5 sccm; ~6 sccm; and ~20 sccm respectively).

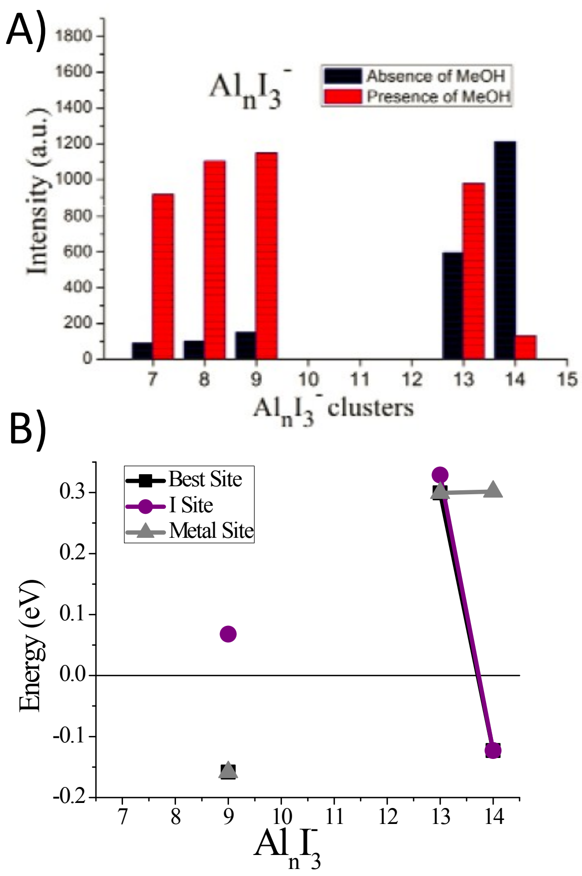

Figure S3. A) Ionic intensities for $Al_nI_3^-$ at the absence and presence of methanol, where the intensity values correspond to the integral areas of the correlated peaks of Figure 1. B) The calculated E_{TS} for the cleavage of the O-H bond of methanol for $Al_nI_3^-$. a.u. = arbitrary units.

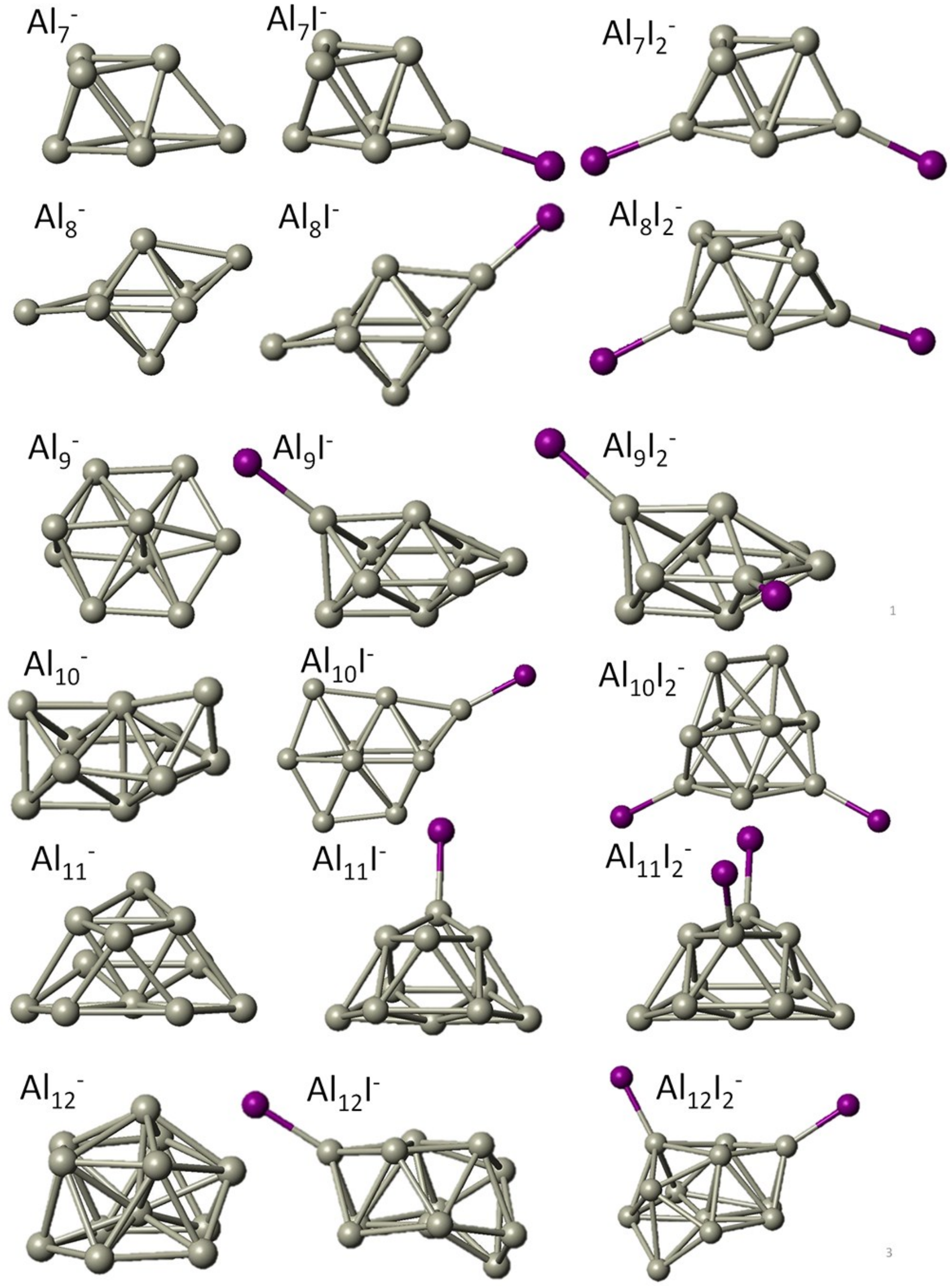

Figure S4. The optimized lowest-energy structures of $Al_{7\text{-}12}I_{0\text{-}2}^{-}$.

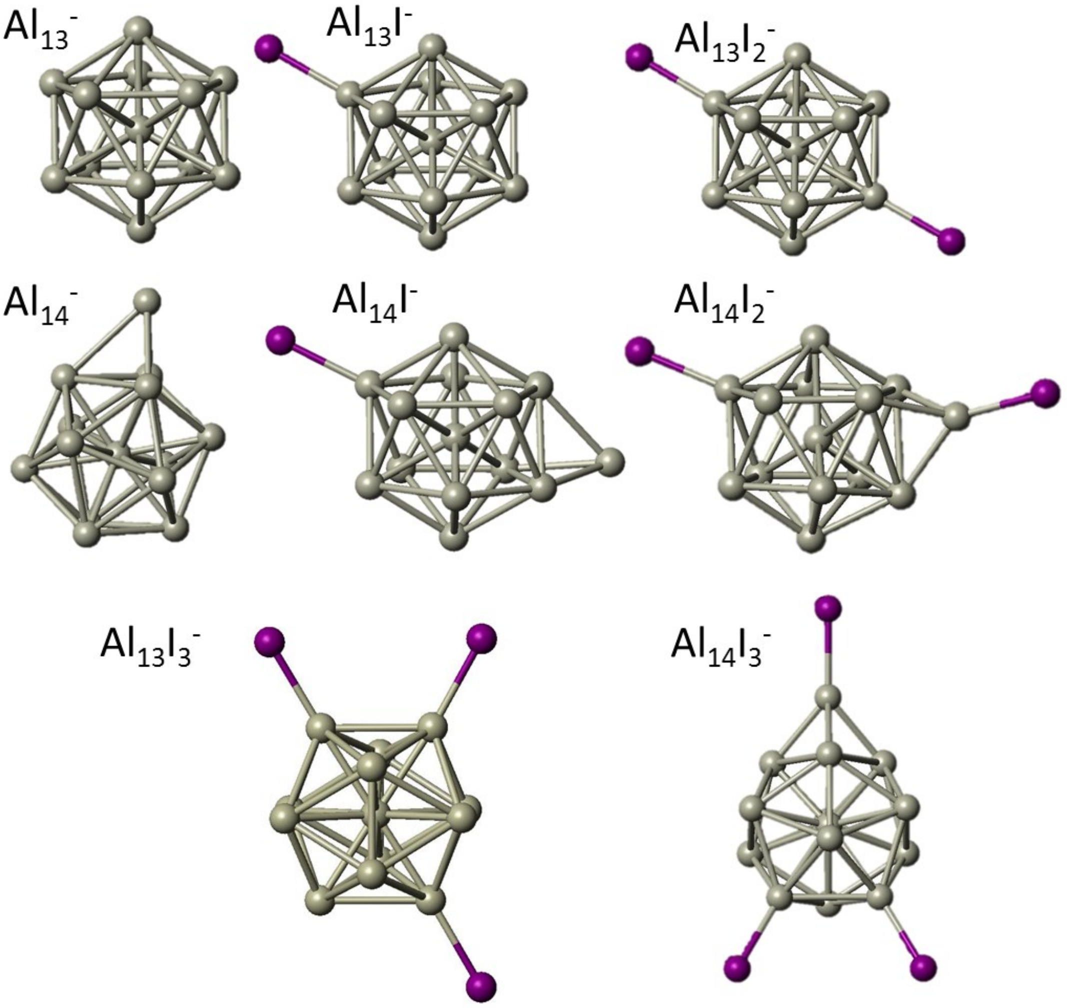

Figure S5. The lowest-energy **s**tructures of $Al_{13,14}I_{0\text{-}3}^-$.

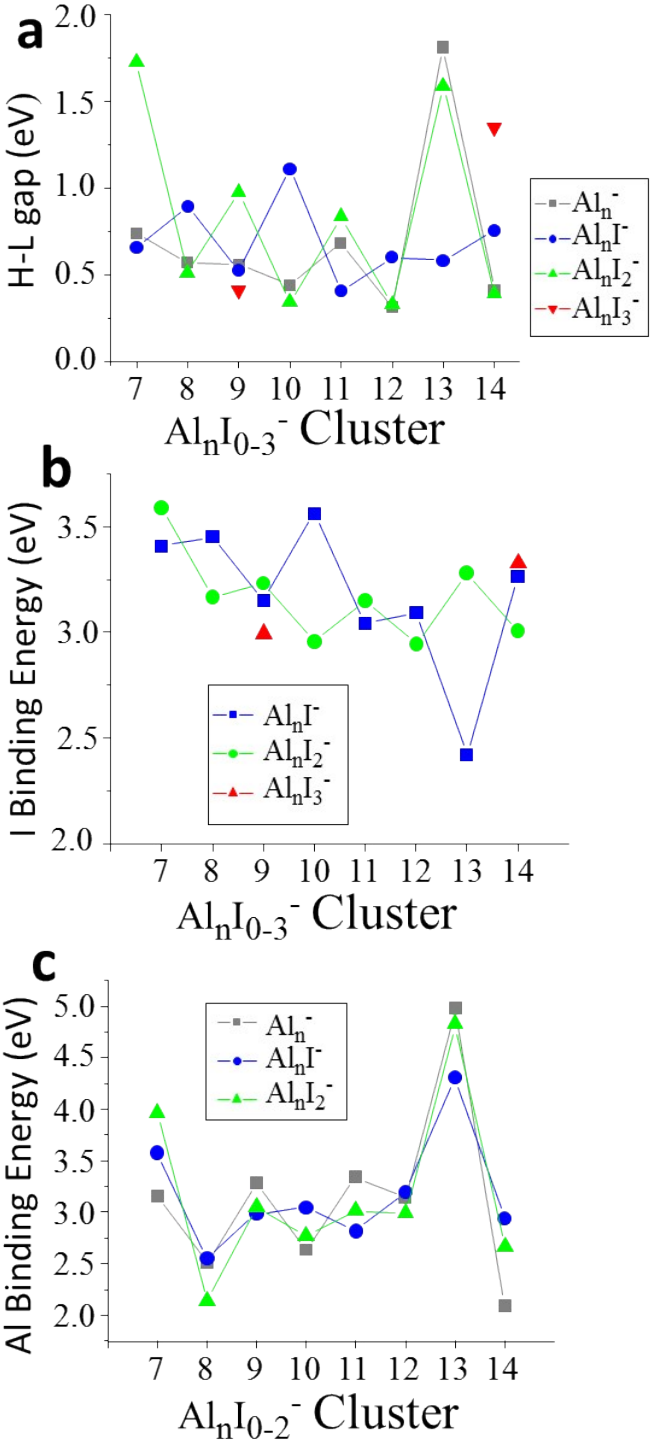

Figure S6. Binding energy and HOMO-LUMO gaps. A**,** The HOMO-LUMO gaps of $Al_nI_m^-$. B, Iodine binding energies of $Al_nI_m^-$ clusters. C, Aluminum binding energies for $Al_nI_{0-2}^-$. n=7-14, m=0-2.

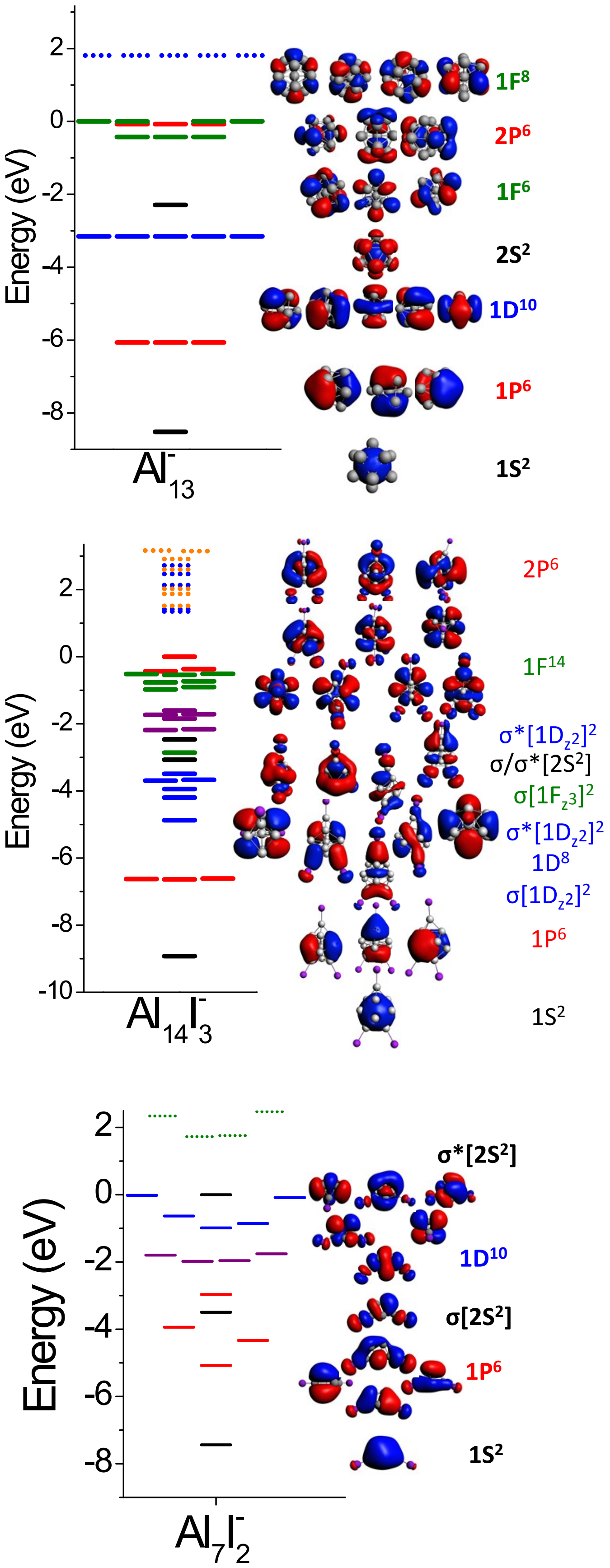

Figure S7. Molecular orbital plot and molecular orbitals of Al_{13}^-, $Al_{14}I_3^-$, and $Al_7I_2^-$. The electronic shell of the orbitals have been indicated.

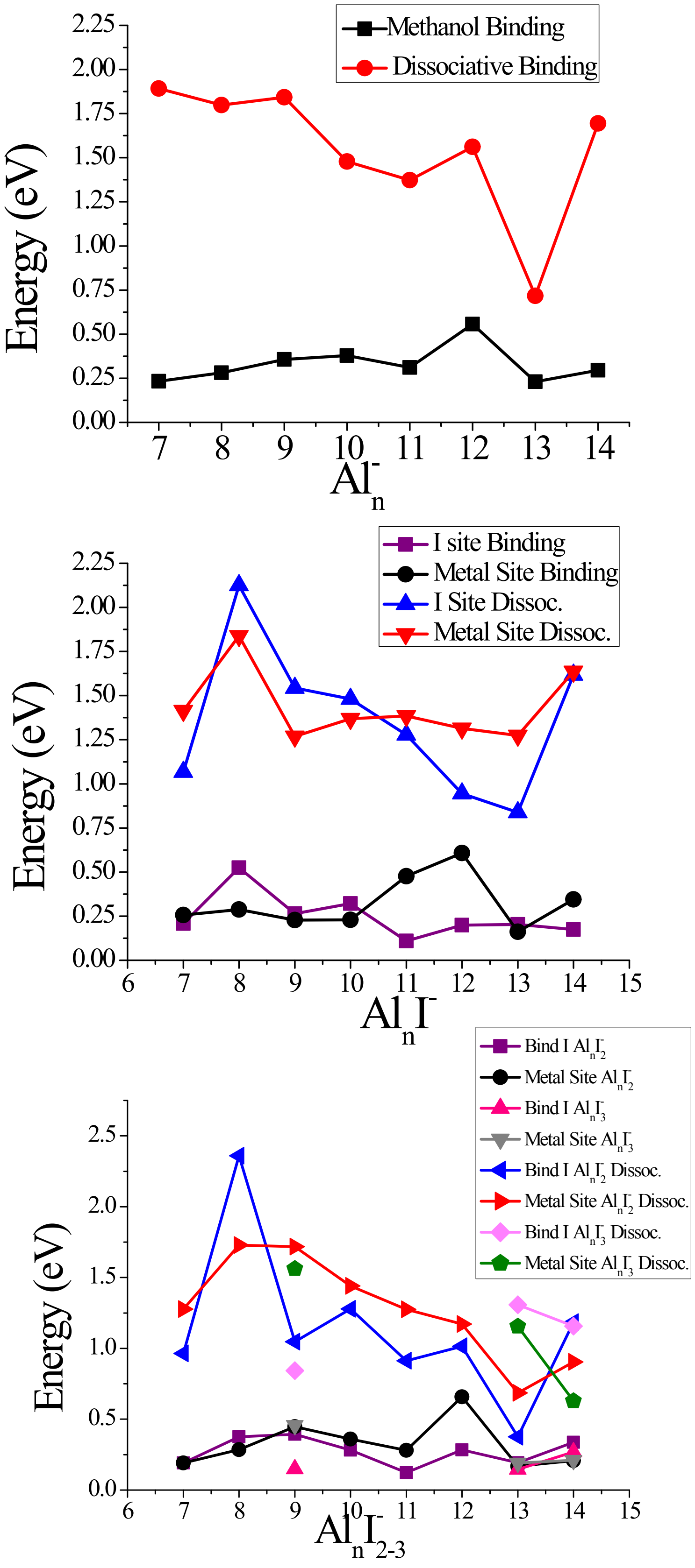

Figure S8. Binding energy of methanol both with the O-H bond intact and dissociative binding, for $Al_nI_{0-3}^-$. n=7-14, and at both the I site and the metal site.

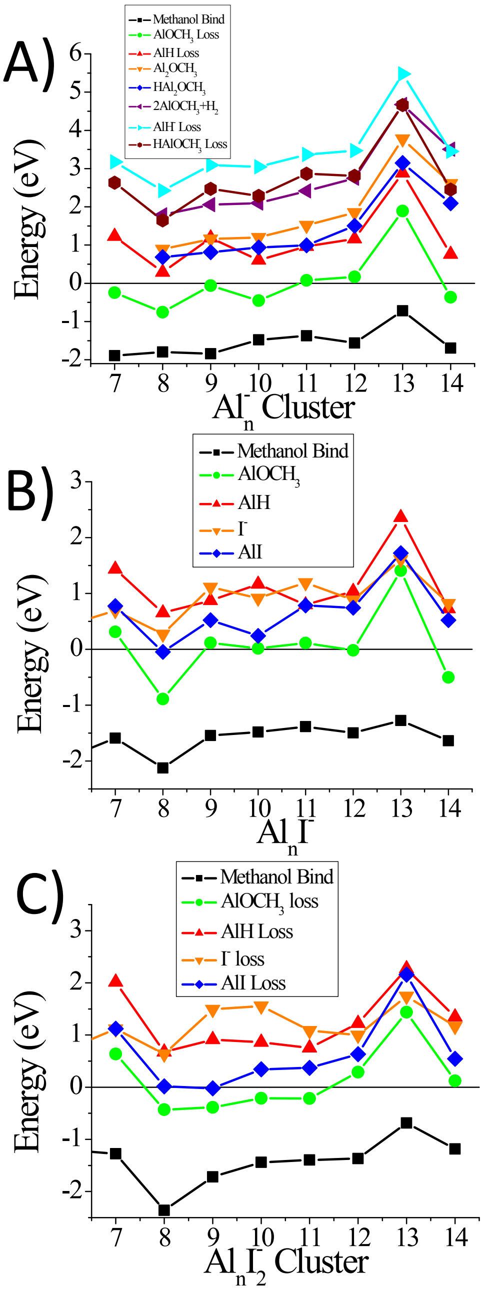

Figure S9. The calculated reaction energy for the dissociative binding of methanol, and the dissociative binding of methanol followed by the loss of the fragment given in the legend. A negative energy implies that the reaction is exothermic. The reactions are as follows:

$Al_n^- + CH_3OH \rightarrow Al_{n-1}H^- + AlOCH_3$ (1)

$Al_n^- + CH_3OH \rightarrow Al_{n-1}HOCH_3^- + AlH$ (2)

$Al_n^- + CH_3OH \rightarrow Al_{n-2}H^- + Al_2OCH_3$ (3)

$Al_n^- + CH_3OH \rightarrow Al_{n-2}^- + HAl_2OCH_3$ (4)

$Al_n^- + 2CH_3OH \rightarrow Al_{n-2}^- + 2AlOCH_3 + H_2$ (5)

$Al_n^- + CH_3OH \rightarrow Al_{n-1}OCH_3 + AlH^-$ (6)

$Al_n^- + CH_3OH \rightarrow Al_{n-1} + HAlOCH_3^-$ (7)

$Al_nI^- + CH_3OH \rightarrow HAl_nOCH_3 + I^-$ (8)

$Al_nI^- + CH_3OH \rightarrow HAl_{n-1}OCH_3^- + AlI$ (9)

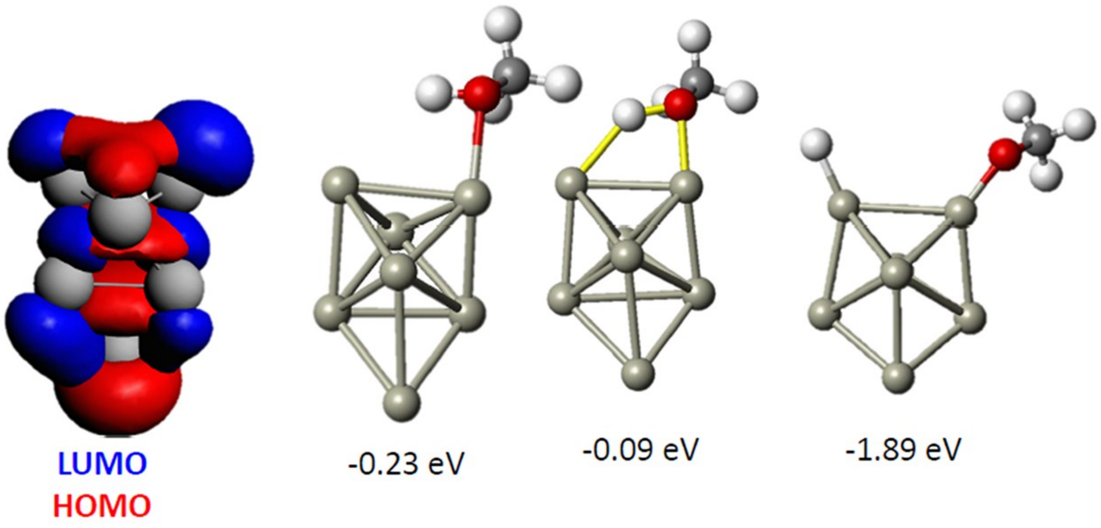

Figure S10. The HOMO and LUMO isosurfaces of Al_7^-, and the binding energy, transition state energy and final state energy for the reaction of methanol with Al_7^-.

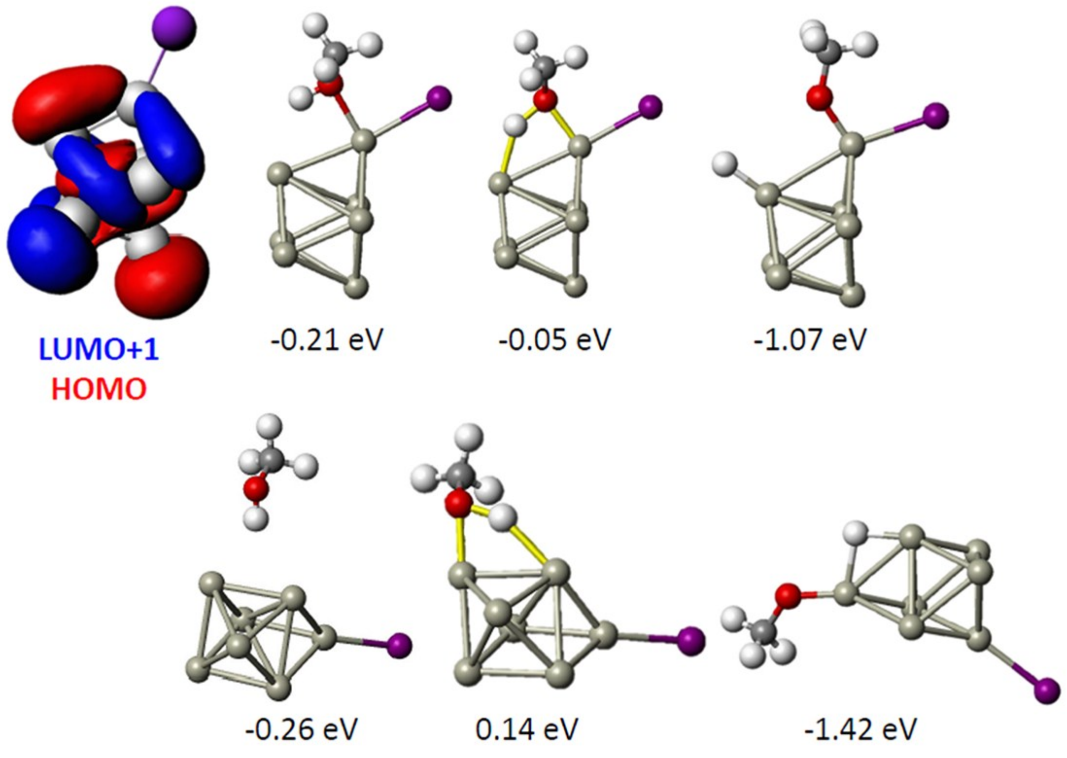

Figure S11. The HOMO and LUMO+1 isosurfaces of Al_7I^-, and the binding energy, transition state energy and final state energy for the reaction of methanol with Al_7I^- at the I site (top row), and metal site (bottom row).

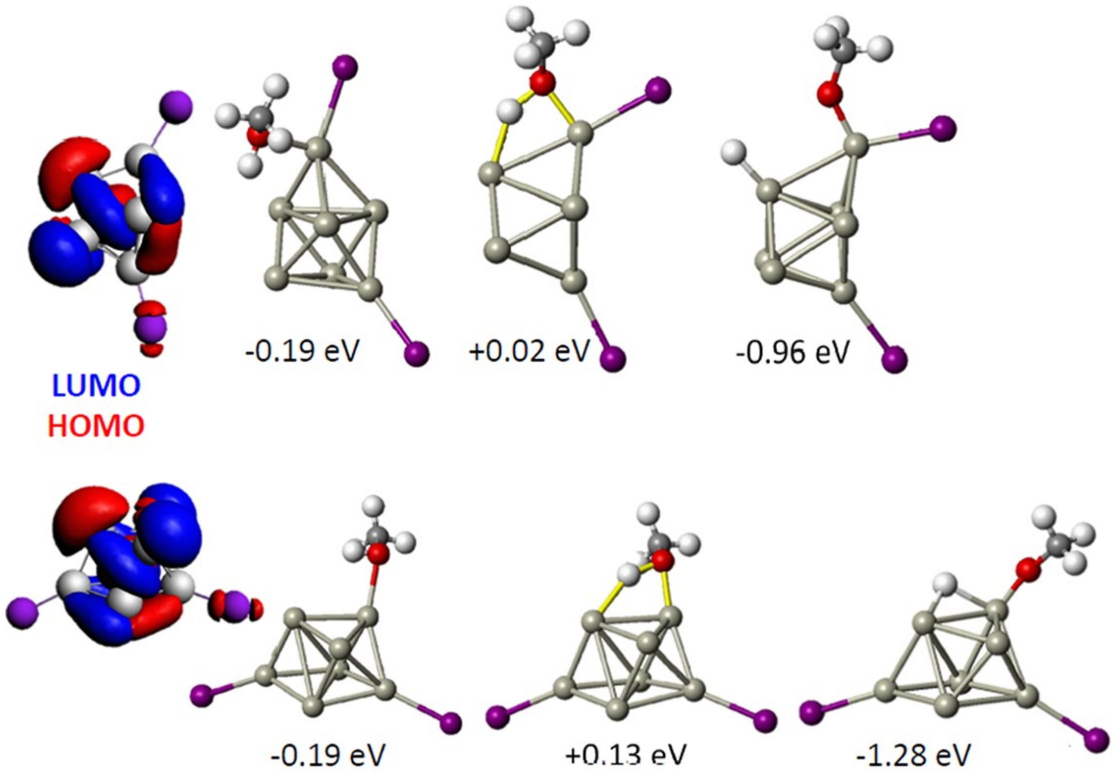

Figure S12. The HOMO and LUMO isosurfaces of $Al_7I_2^-$, and the binding energy, transition state energy and final state energy for the reaction of methanol with $Al_7I_2^-$ at the I site (top row), and metal site (bottom row).

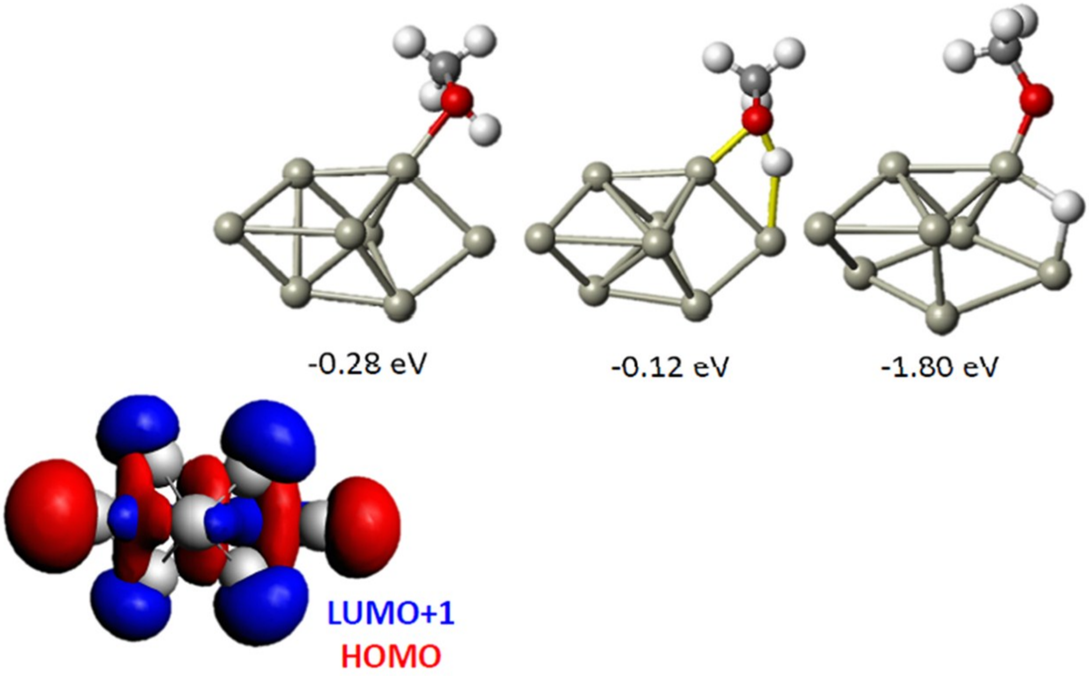

Figure S13. The HOMO and LUMO+1 isosurfaces of Al_8^-, and the binding energy, transition state energy and final state energy for the reaction of methanol with Al_8^-.

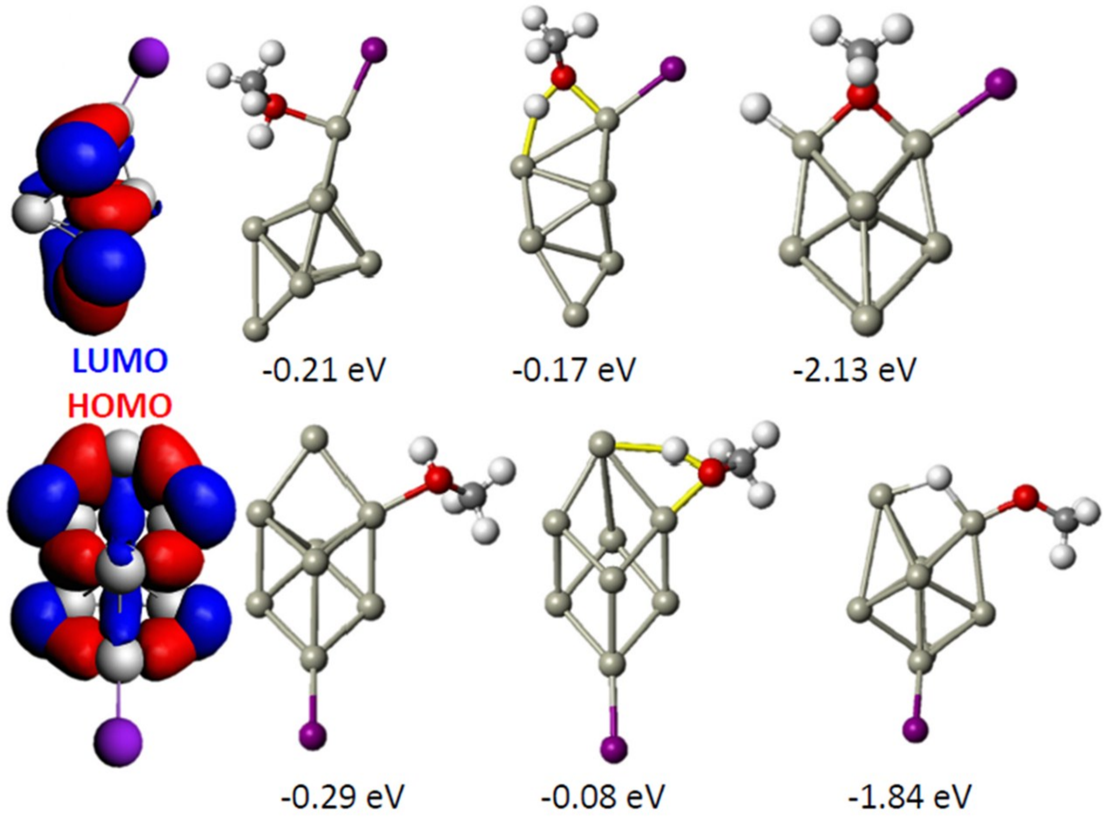

Figure S14. The HOMO and LUMO isosurfaces of Al_8I^-, and the binding energy, transition state energy and final state energy for the reaction of methanol with Al_8I^- at the I site (top row), and metal site (bottom row).

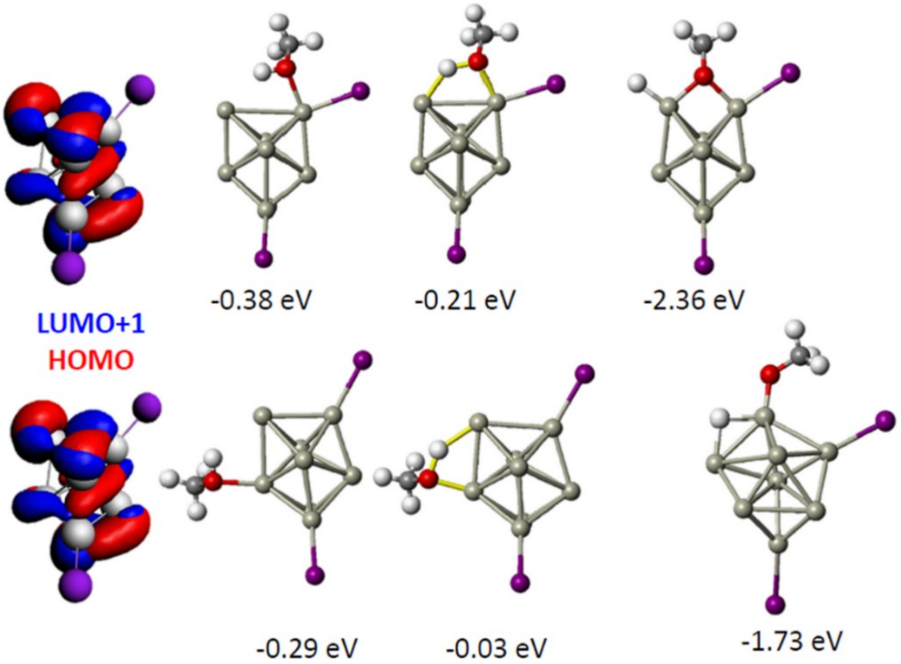

Figure S15. The HOMO and LUMO+1 isosurfaces of $Al_8I_2^-$, and the binding energy, transition state energy and final state energy for the reaction of methanol with $Al_7I_2^-$ at the I site (top row), and metal site (bottom row).

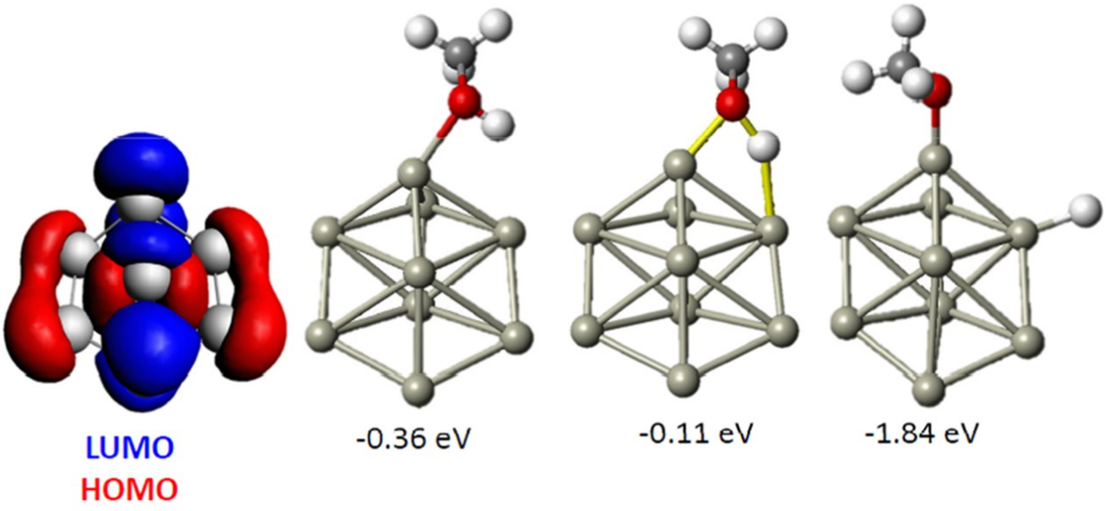

Figure S16. The HOMO and LUMO isosurfaces of Al_9^-, and the binding energy, transition state energy and final state energy for the reaction of methanol with Al_9^-.

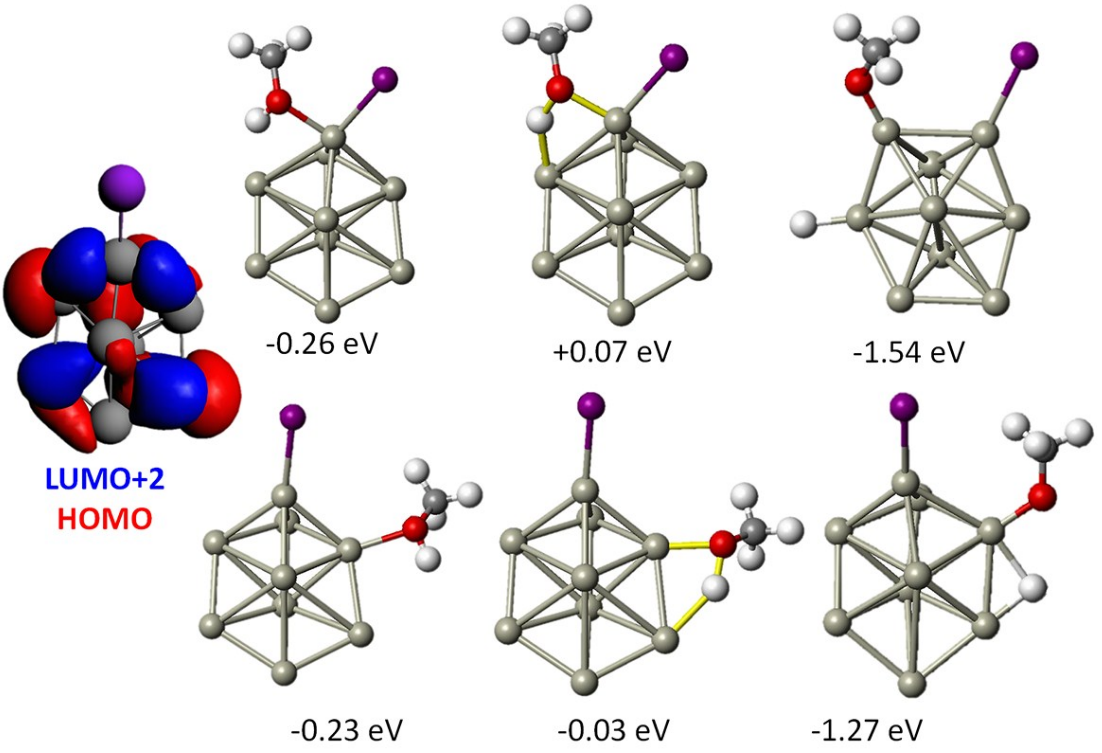

Figure S17. The HOMO and LUMO+2 isosurfaces of Al_9I^-, and the binding energy, transition state energy and final state energy for the reaction of methanol with Al_9I^- at the I site (top row), and metal site (bottom row).

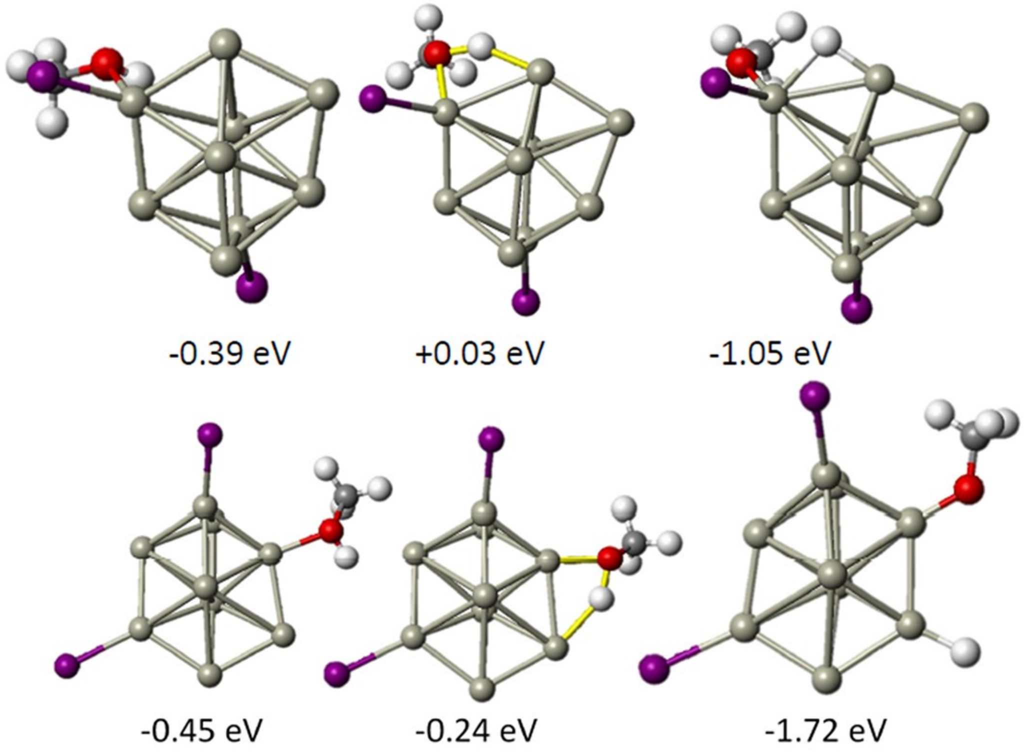

Figure S18. The HOMO and LUMO+2 isosurfaces of $Al_9I_2^-$, and the binding energy, transition state energy and final state energy for the reaction of methanol with $Al_9I_2^-$ at the I site (top row), and metal site (bottom row).

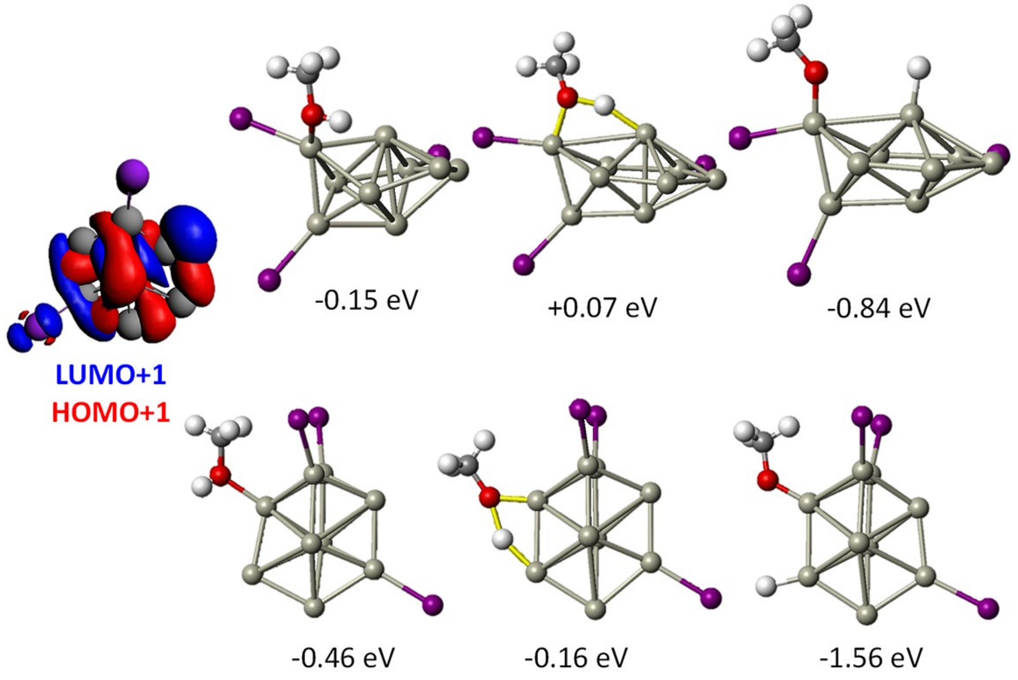

Figure S19. The HOMO+1 and LUMO+1 isosurfaces of the ground state of $Al_9I_3^-$, and the binding energy, transition state energy and final state energy for the reaction of methanol with $Al_9I_3^-$ at the I site (top row), and metal site (bottom row).

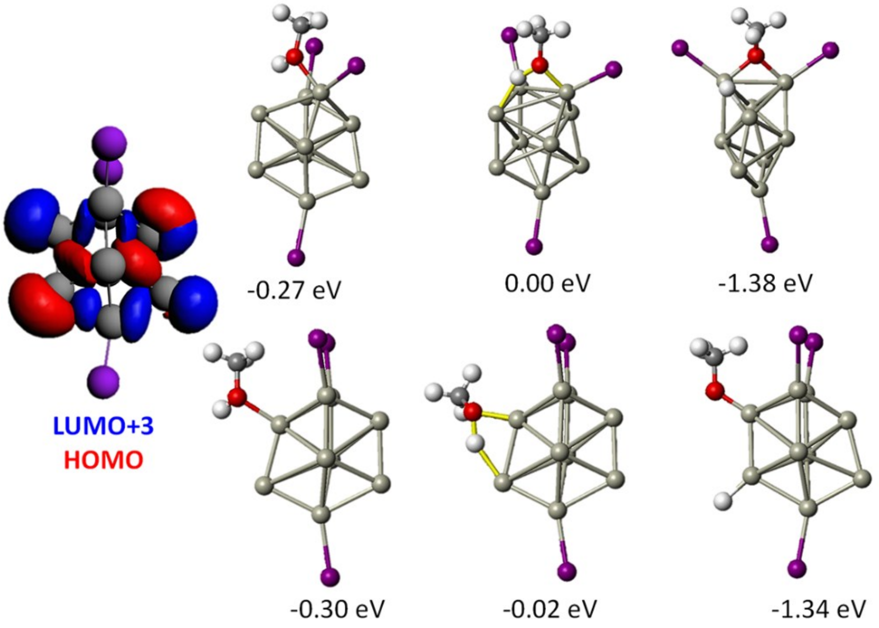

Figure S20. The HOMO and LUMO+3 isosurfaces of the second isomer of $Al_9I_3^-$, and the binding energy, transition state energy and final state energy for the reaction of methanol with the second isomer $Al_9I_3^-$ at the I site (top row), and metal site (bottom row). The energy is +0.03 eV higher than the isomer from S16.

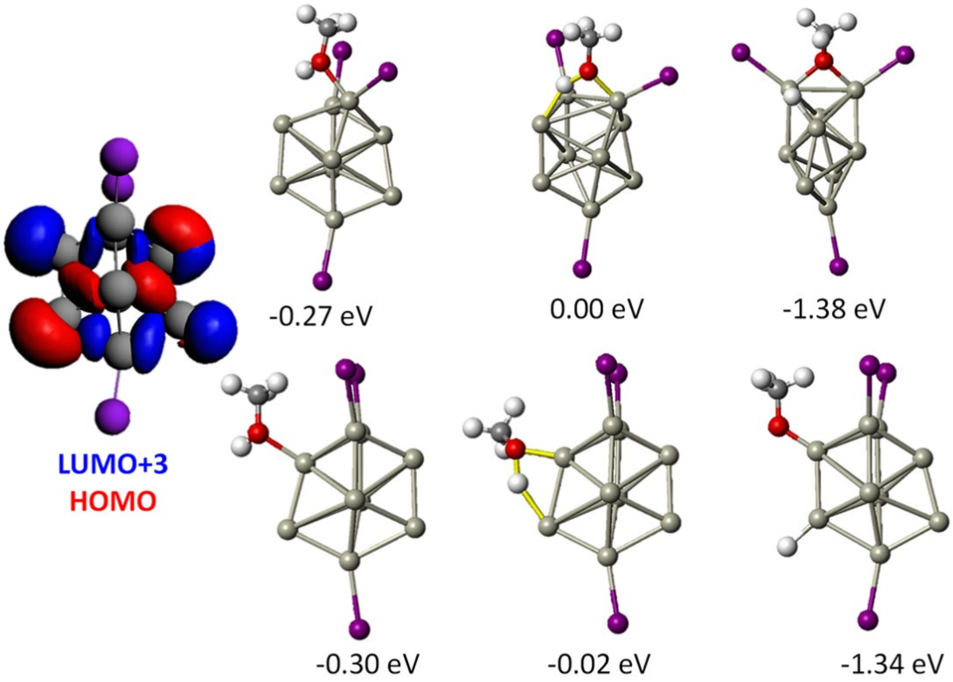

Figure S21. The HOMO and LUMO isosurfaces of the third isomer of $Al_9I_3^-$, and the binding energy, transition state energy and final state energy for the reaction of methanol with the third isomer $Al_9I_3^-$ at the I site (top row), and metal site (bottom row). The energy is +0.07 eV higher than the isomer from S16.

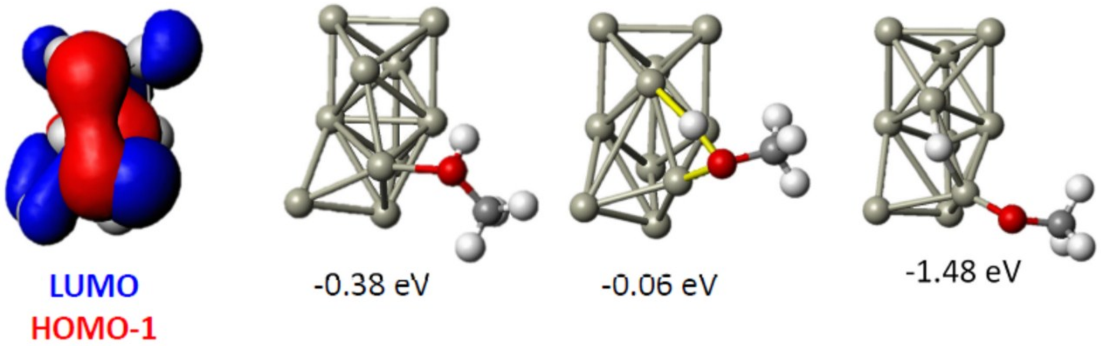

Figure S22. The HOMO and LUMO+1 isosurfaces of Al_{10}^-, and the binding energy, transition state energy and final state energy for the reaction of methanol with Al_{10}^-.

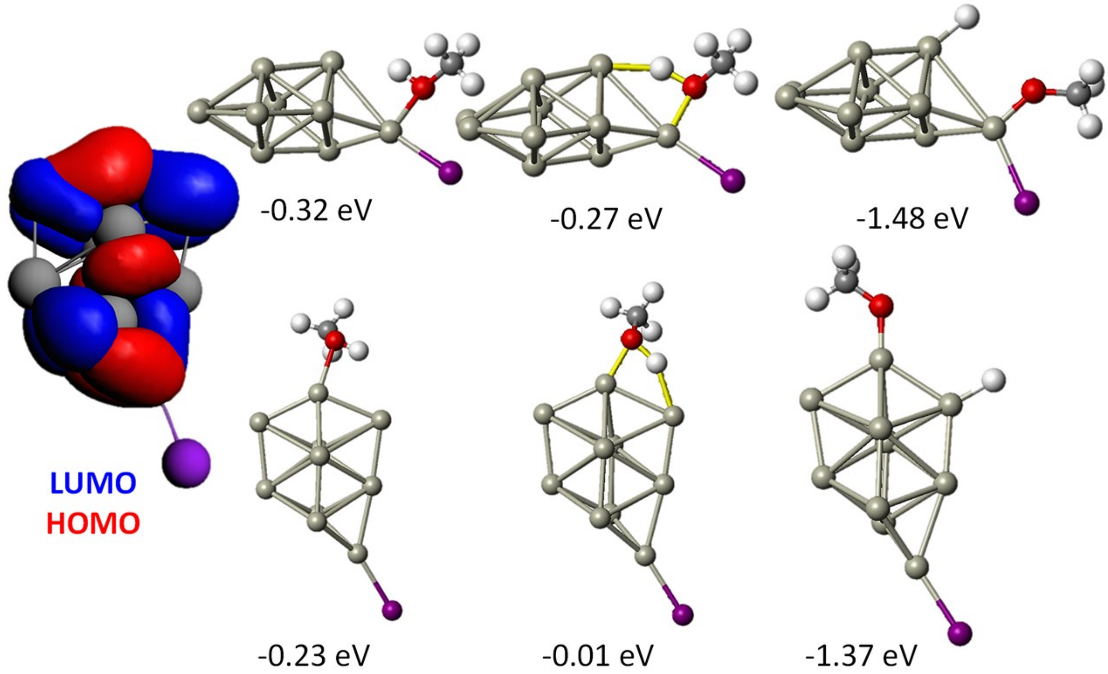

Figure S23. The HOMO and LUMO isosurfaces of $Al_{10}I^-$, and the binding energy, transition state energy and final state energy for the reaction of methanol with $Al_{10}I^-$ at the I site (top row), and metal site (bottom row).

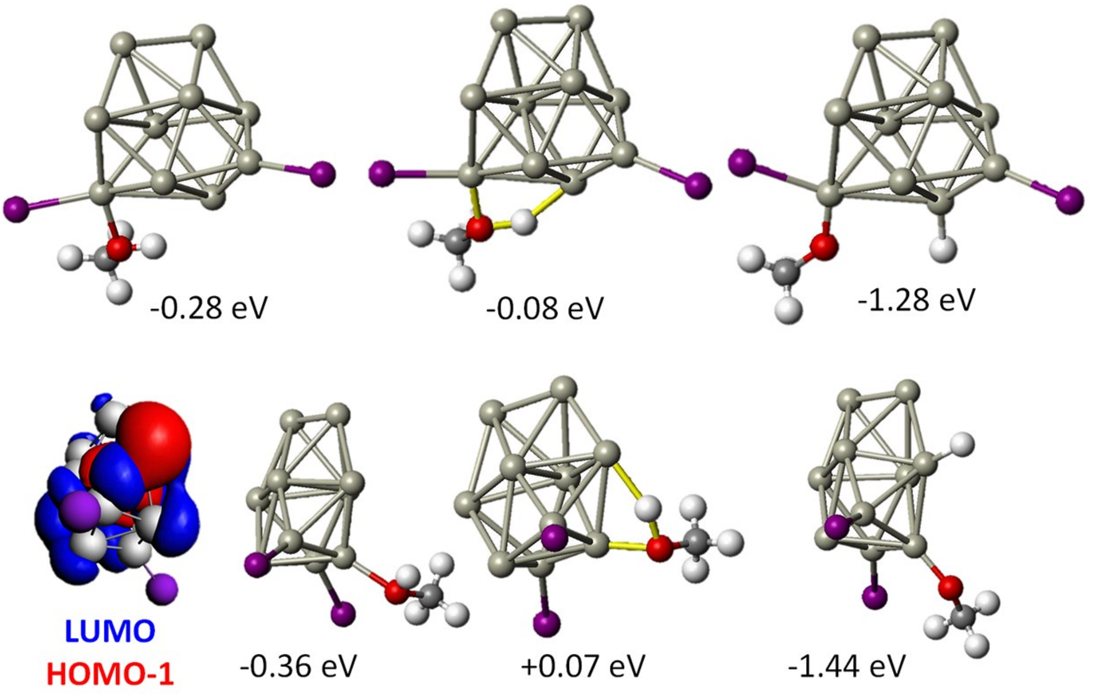

Figure S24. The HOMO-1 and LUMO isosurfaces of $Al_{10}I_2^-$, and the binding energy, transition state energy and final state energy for the reaction of methanol with $Al_{10}I_2^-$ at the I site (top row), and metal site (bottom row).

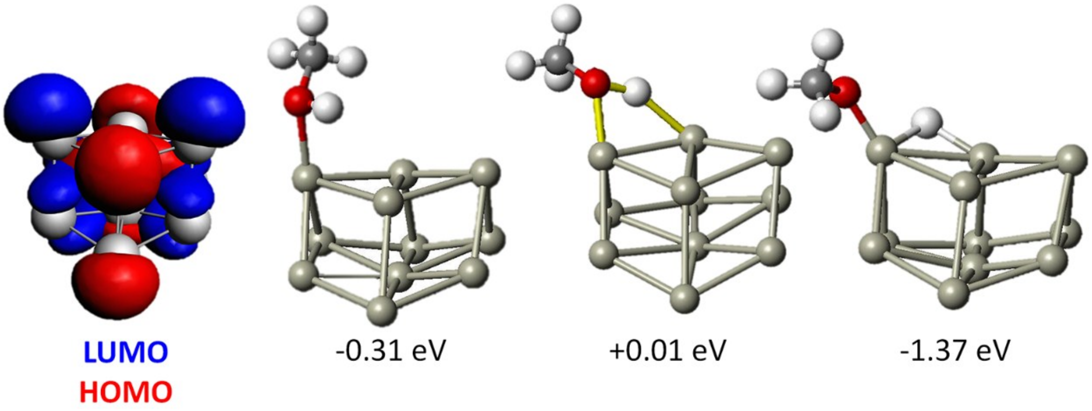

Figure S25. The HOMO and LUMO isosurfaces of Al_{11}^-, and the binding energy, transition state energy and final state energy for the reaction of methanol with Al_{11}^-.

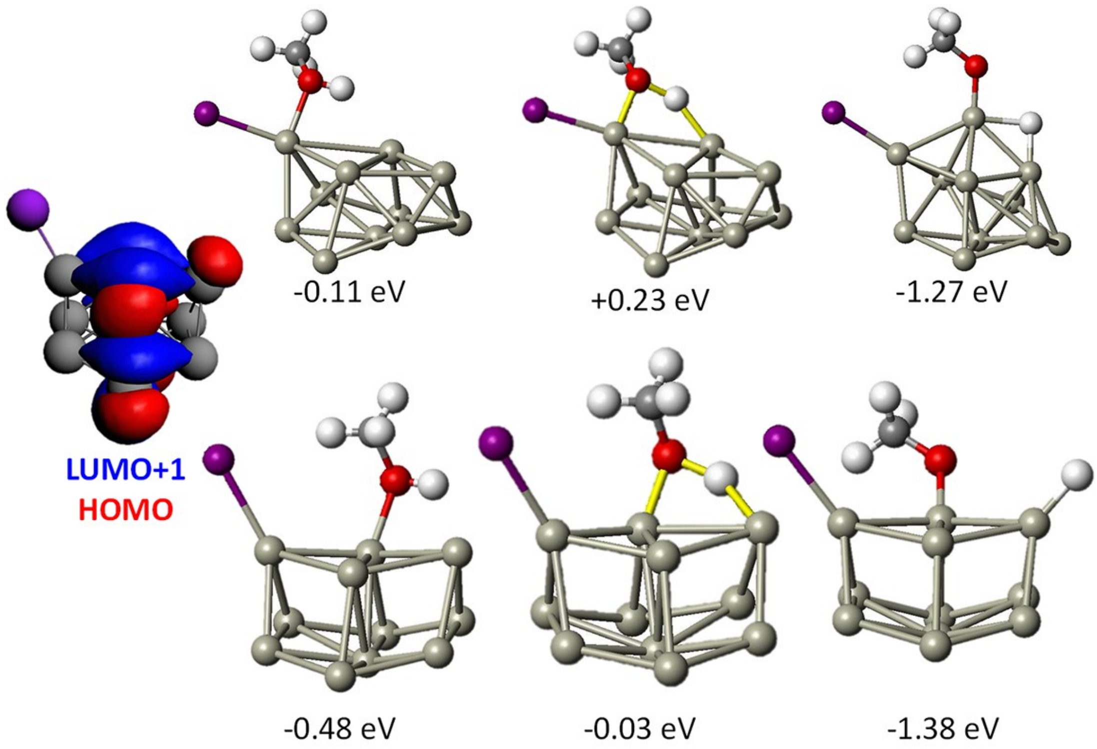

Figure S26. The HOMO and LUMO+1 isosurfaces of $Al_{11}I^-$, and the binding energy, transition state energy and final state energy for the reaction of methanol with $Al_{11}I^-$ at the I site (top row), and metal site (bottom row).

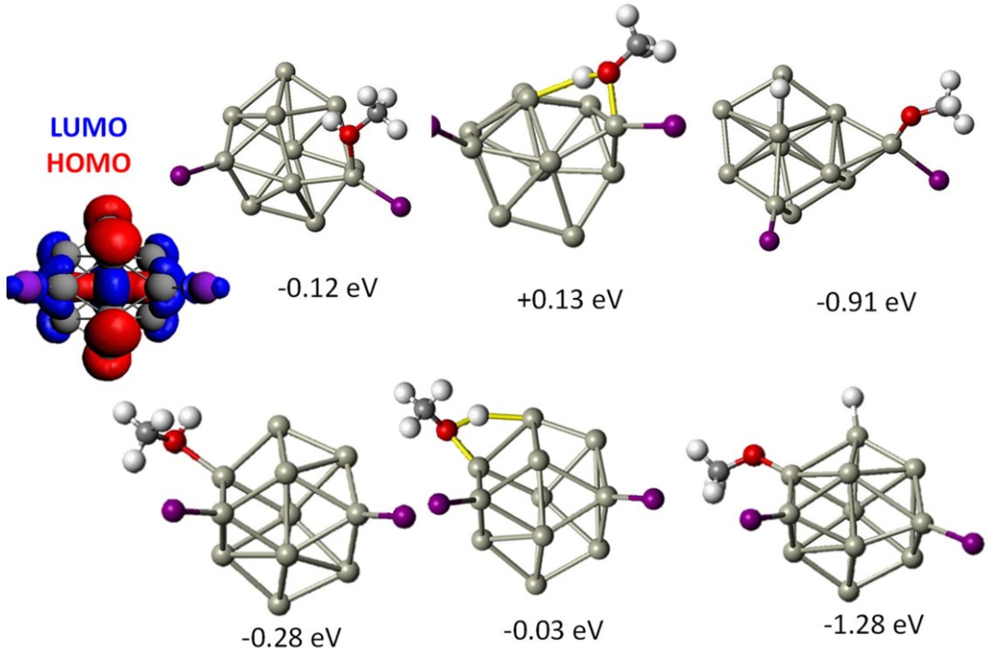

Figure S27. The HOMO and LUMO isosurfaces of $Al_{11}I_2^-$, and the binding energy, transition state energy and final state energy for the reaction of methanol with $Al_{11}I_2^-$ at the I site (top row), and metal site (bottom row).

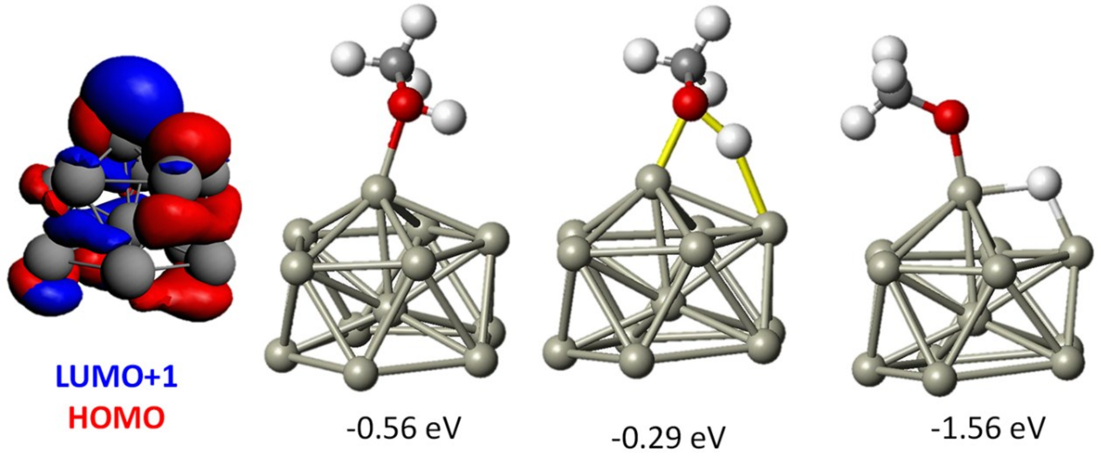

Figure S28. The HOMO and LUMO+1 isosurfaces of Al_{12}^{-}, and the binding energy, transition state energy and final state energy for the reaction of methanol with Al_{12}^{-}.

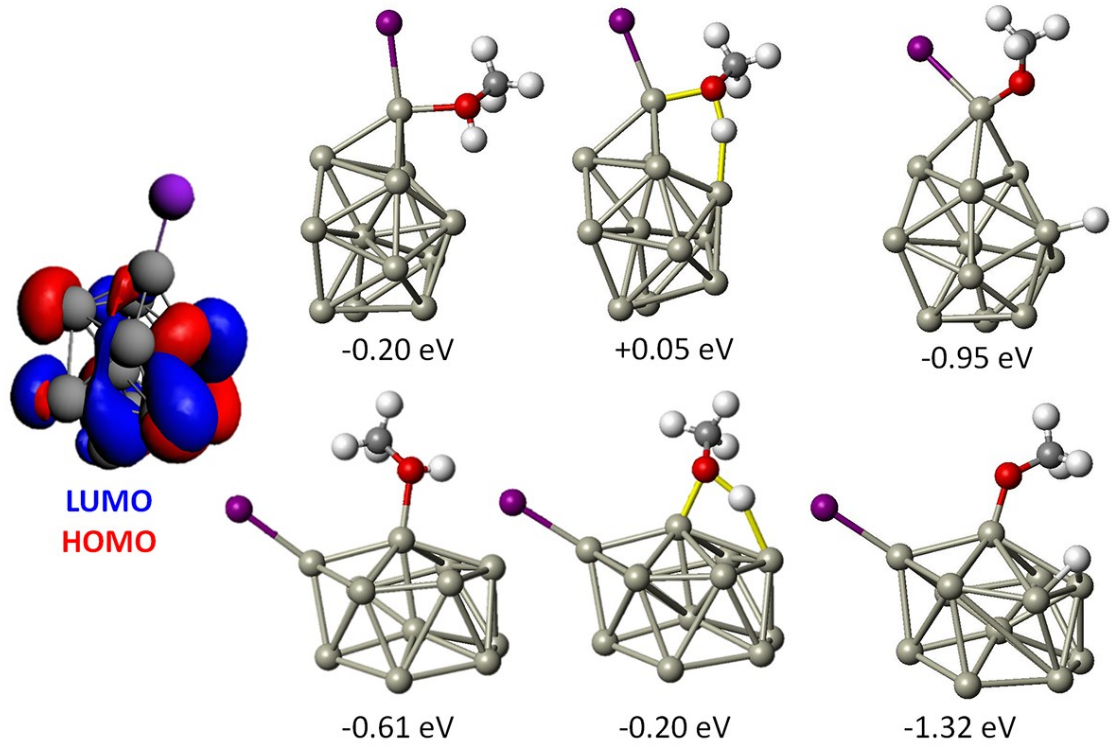

Figure S29. The HOMO and LUMO isosurfaces of $Al_{12}I^{-}$, and the binding energy, transition state energy and final state energy for the reaction of methanol with $Al_{12}I^{-}$ at the I site (top row), and metal site (bottom row).

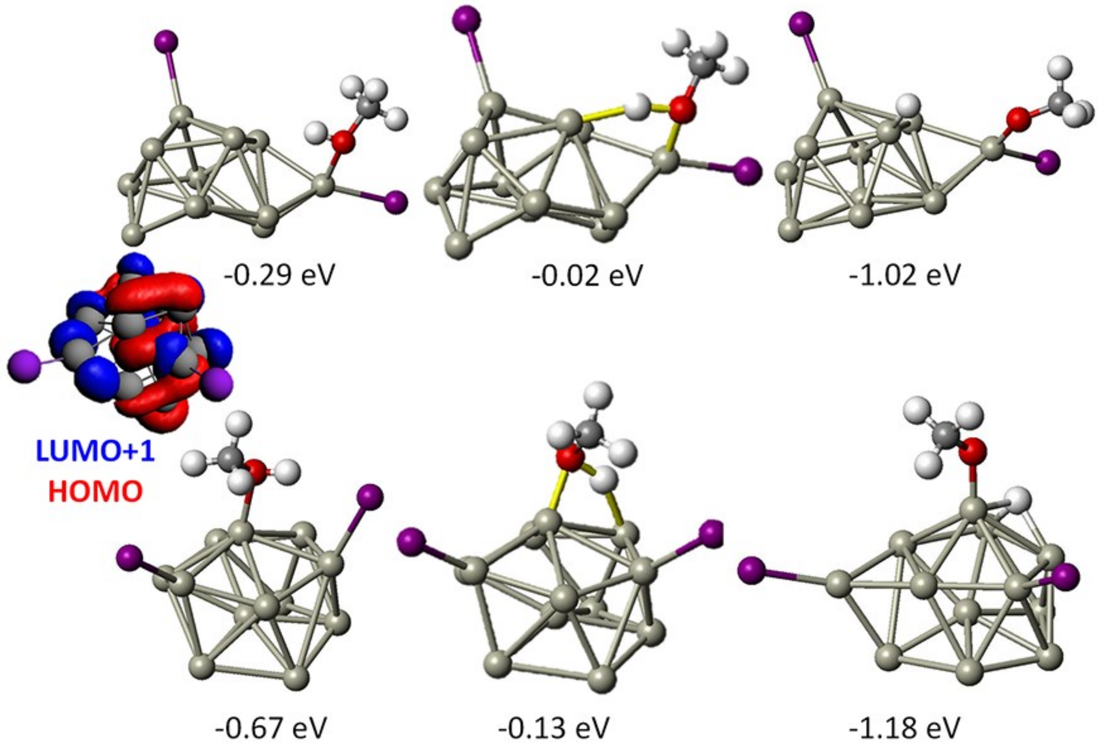

Figure S30. The HOMO and LUMO+1 isosurfaces of $Al_{12}I_2^-$, and the binding energy, transition state energy and final state energy for the reaction of methanol with $Al_{12}I_2^-$ at the I site (top row), and metal site (bottom row).

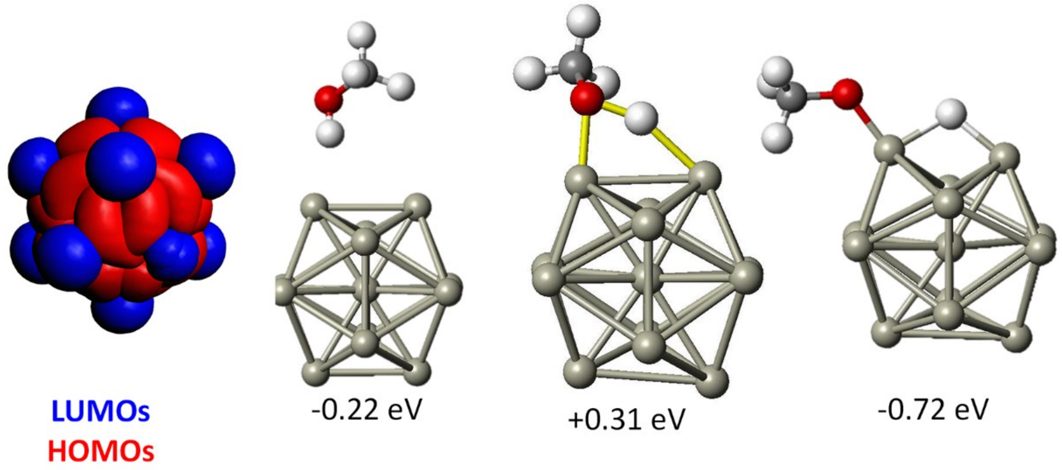

Figure S31. The HOMO-HOMO-3 and LUMO-LUMO+3 isosurfaces of Al_{13}^-, and the binding energy, transition state energy and final state energy for the reaction of methanol with Al_{13}^-.

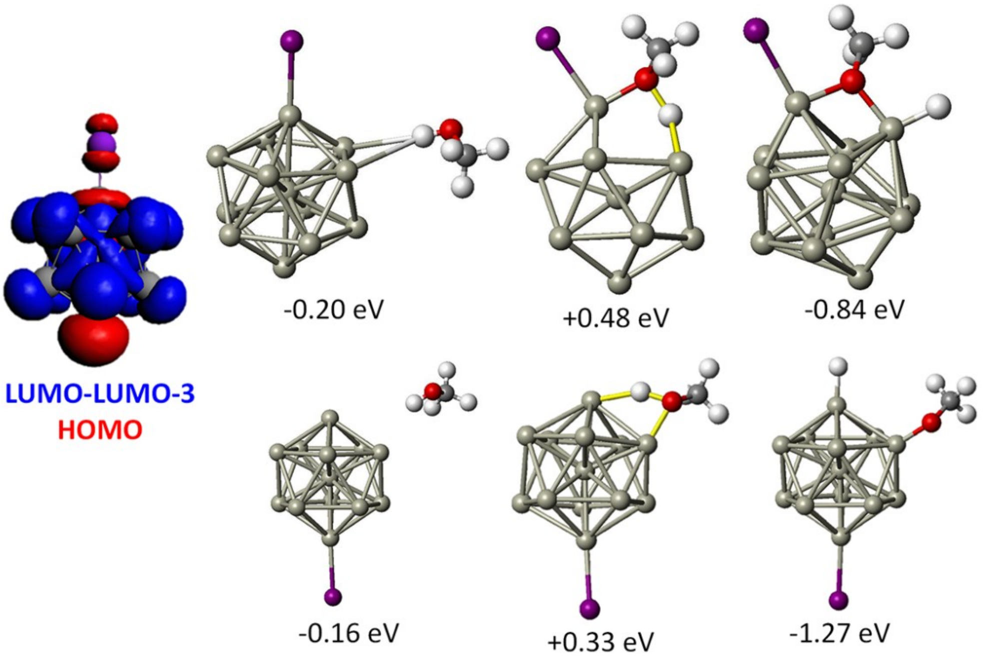

Figure S32. The HOMO and LUMO-LUMO-3 isosurfaces of $Al_{13}I^{-}$, and the binding energy, transition state energy and final state energy for the reaction of methanol with $Al_{13}I^{-}$ at the I site (top row), and metal site (bottom row).

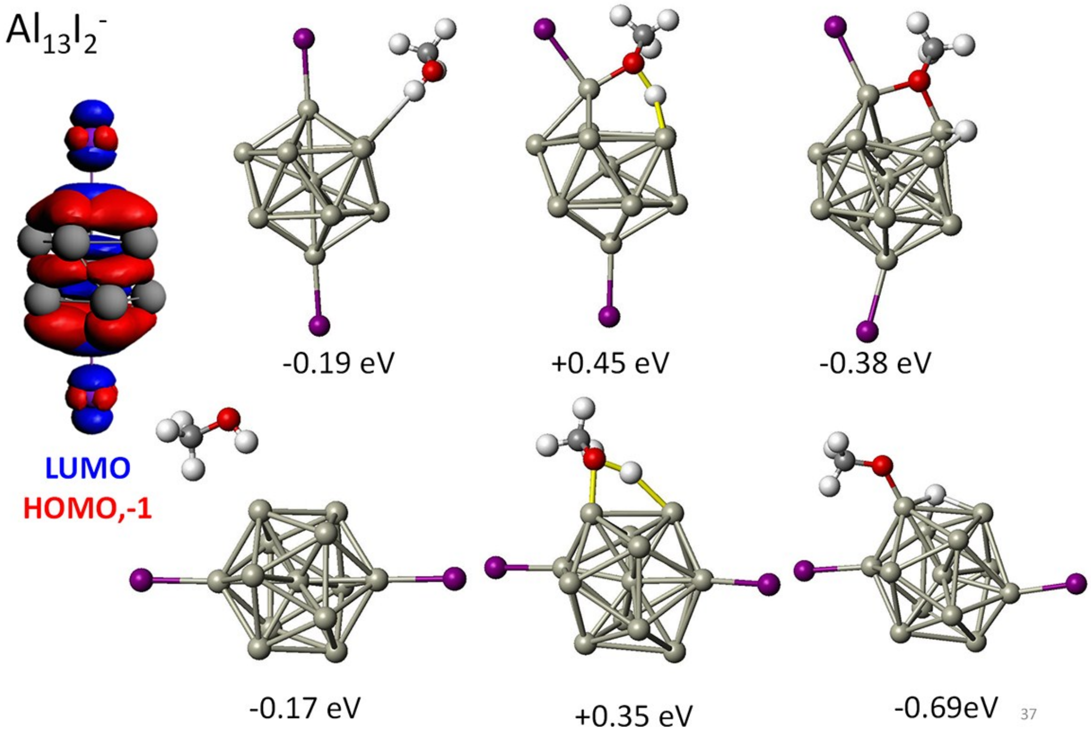

Figure S33. The HOMO/HOMO-1 and LUMO isosurfaces of $Al_{13}I_2^{-}$, and the binding energy, transition state energy and final state energy for the reaction of methanol with $Al_{13}I_2^{-}$ at the I site (top row), and metal site (bottom row).

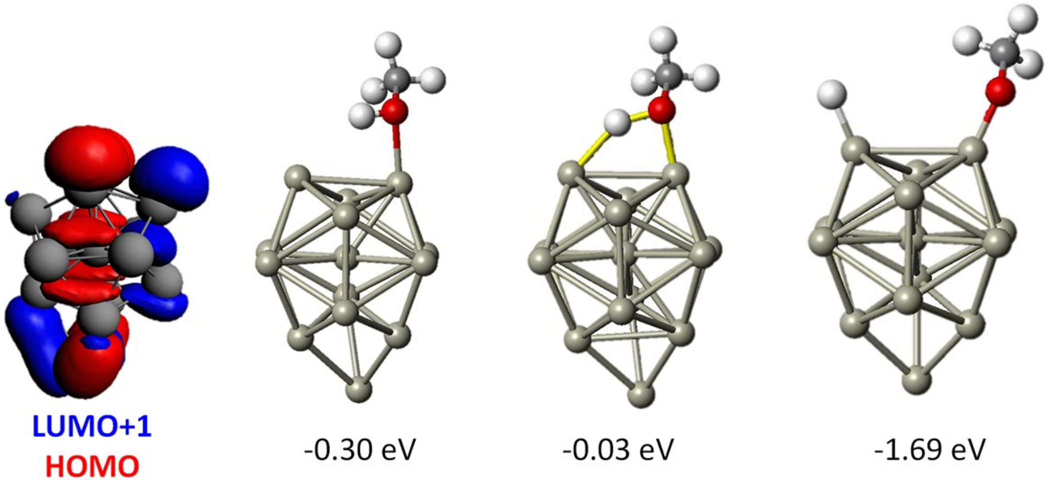

Figure S34. The HOMO and LUMO+1 isosurfaces of Al_{14}^{-}, and the binding energy, transition state energy and final state energy for the reaction of methanol with Al_{14}^{-}.

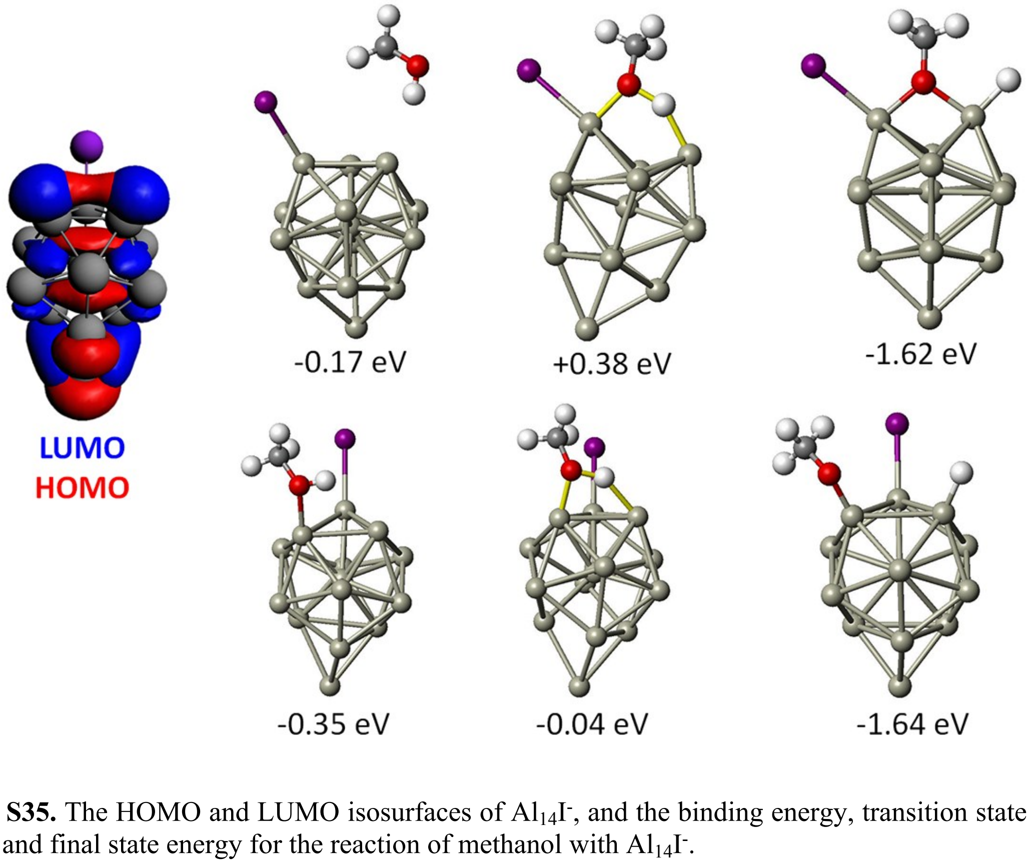

Figure S35. The HOMO and LUMO isosurfaces of $Al_{14}I^{-}$, and the binding energy, transition state energy and final state energy for the reaction of methanol with $Al_{14}I^{-}$.

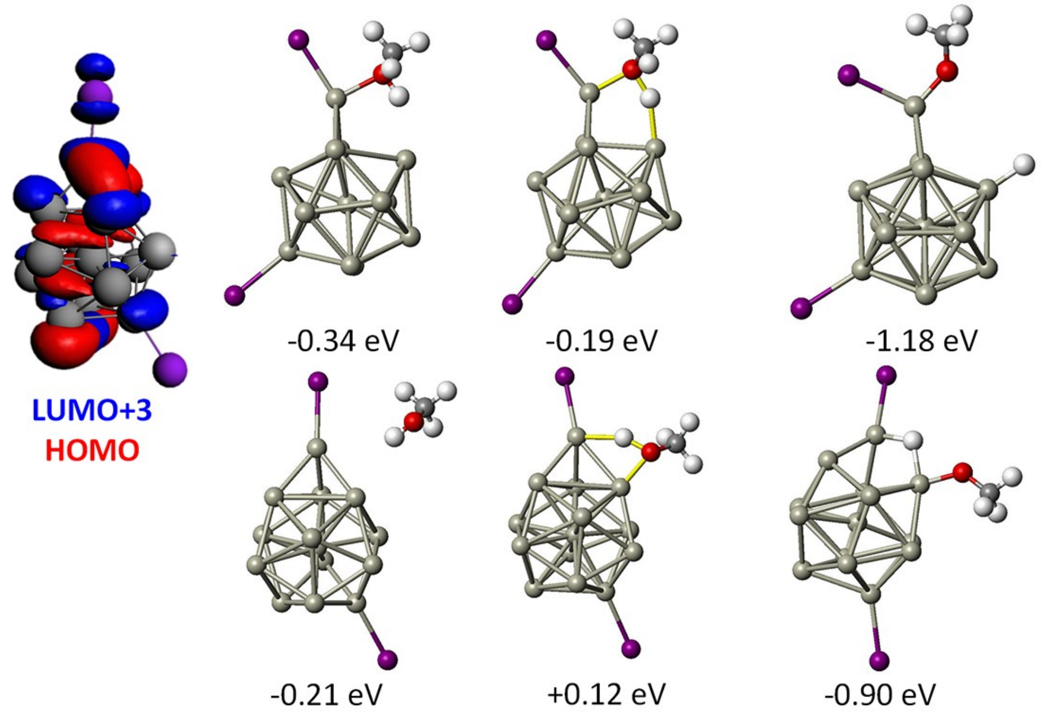

Figure S36. The HOMO and LUMO+3 isosurfaces of $Al_{14}I_2^-$, and the binding energy, transition state energy and final state energy for the reaction of methanol with $Al_{14}I_2^-$.

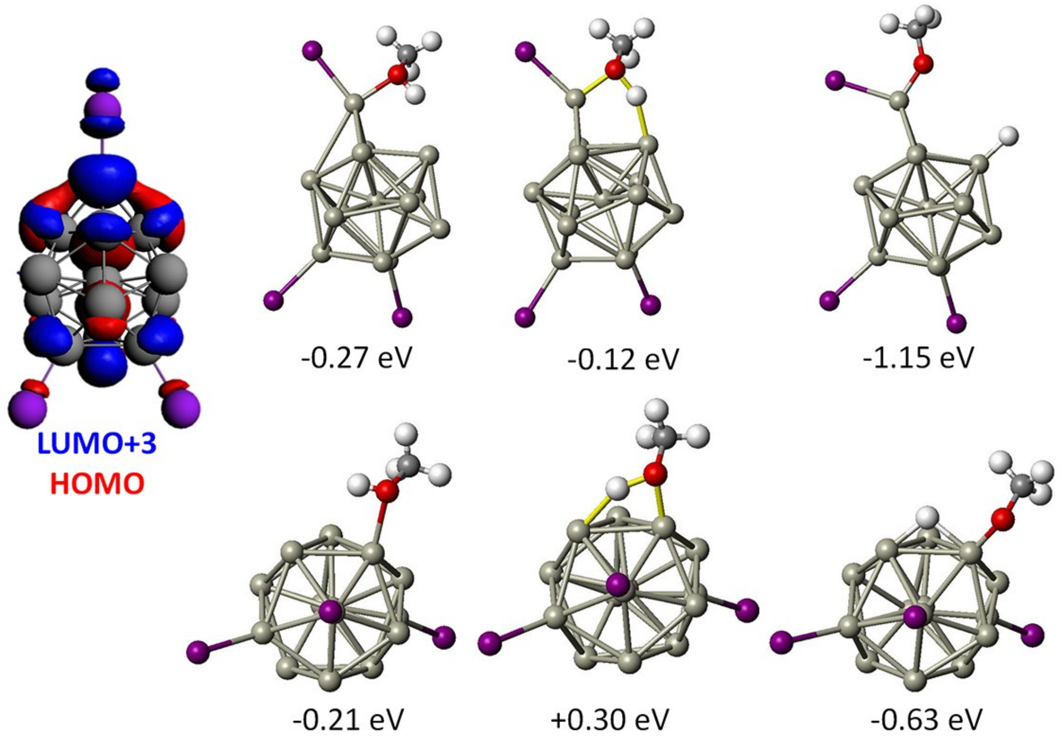

Figure S37. The HOMO and LUMO+3 isosurfaces of $Al_{14}I_3^-$, and the binding energy, transition state energy and final state energy for the reaction of methanol with $Al_{14}I_3^-$.

#Attachment:

The coordinates in Angstroms, and energy of the clusters. All energies are relative to the spin restricted atoms.

Al_7^-

-19.2574 eV

Al	-1.514056	-0.410724	1.50919
Al	0.584475	1.081252	2.036582
Al	-1.97884	-1.201287	-1.118007
Al	-0.2606	2.932333	0.240441
Al	0.18144	0.442031	-0.483139
Al	-1.917644	2.101763	2.197637
Al	-2.363635	1.442332	-0.314695

Al_7I^-

-22.81898 eV

Al	-2.592882	-0.025803	1.835074
Al	-0.109253	0.537132	2.006241
Al	-1.54714	-0.639772	-0.507822
Al	-0.412785	2.249789	-0.164251
Al	0.93853	-0.023213	-0.363749
Al	-1.748599	2.662566	2.12554
Al	-2.887133	1.617827	-0.242935
I	3.300863	-0.725625	-1.230599

$Al_7I_2^-$

-26.57126 eV

Al	-1.407386	1.605562	-0.020426
I	-2.053744	4.130229	-0.213717
Al	0.138679	0.023775	1.411907
Al	-2.382544	-0.374173	1.622875
Al	-1.386384	-2.469295	0.383652
Al	1.11783	-2.073033	0.101013
I	3.418904	-3.291992	-0.002967
Al	-2.634085	-0.643281	-1.040617
Al	-0.127471	-0.249793	-1.383219

Al_8^-

-21.948444 eV

Al	0.096368	-0.034538	2.495874
Al	1.916508	-1.900523	2.203089
Al	4.389422	-2.627967	2.348916
Al	3.603244	-0.761885	0.521476
Al	2.535148	0.90591	2.267454
Al	5.083147	0.518198	2.42095
Al	3.635407	-0.852119	4.152676
Al	6.349167	-1.381544	1.131124

Al_8I^-

-25.55455204 eV

Al	0.187922	-0.066045	2.375175
Al	1.977527	-1.992599	2.144359
Al	4.469565	-2.572726	2.526874
Al	3.635718	-0.755814	0.558129
Al	2.612487	0.96663	2.227403
Al	5.11988	0.452255	2.606692
Al	3.403344	-0.806498	4.176986
Al	6.296162	-1.345813	1.199033
I	8.441749	-1.774132	-0.225054

$Al_8I_2^-$

-28.87738 eV

Al	0.162192	0.100865	0.026477
I	-0.15375	-0.151998	2.586008
Al	2.995189	0.15803	-1.079379
Al	1.668873	0.423885	-3.659978
Al	0.887318	1.959043	-1.686694
Al	-0.760641	-0.750875	-4.308578
Al	-0.469479	1.994883	-3.9459
I	-1.196238	3.812911	-5.643962
Al	-1.424251	0.493423	-2.04381
Al	0.643811	-1.315477	-2.078281

Al_9^-

-25.41556 eV

Al	0.001401	-0.0001	0.008618
Al	0.00143	-0.000465	2.547487

Al	2.56116	-0.003808	0.003225
Al	2.575085	-0.355491	2.516398
Al	1.105231	-2.232451	0.97295
Al	0.987045	-2.581277	3.538505
Al	1.294529	-0.347394	4.987671
Al	1.594343	2.064445	3.858606
Al	1.459275	2.073041	1.27024

Al_9I^-

-28.721076 eV

Al	-0.31138	0.170633	-0.100951
Al	-0.007644	-0.080465	2.834257
Al	2.182462	-0.144284	1.165018
Al	3.587546	-0.403667	3.348639
Al	0.330729	-2.270855	0.64377
Al	1.701218	-2.230724	2.890413
Al	1.476868	-0.384954	4.907277
Al	2.122318	1.804777	3.398702
Al	0.700122	2.140712	1.259052
I	1.245001	-0.678602	7.477504

$Al_9I_2^-$

-32.10856 eV

Al	-0.45886	0.156152	0.259533
Al	-0.182352	-0.269212	2.809671
Al	2.134564	0.021483	-0.079278
Al	2.623335	-0.420216	2.466446
Al	0.879671	-2.128863	0.839702
Al	1.262285	-2.573931	3.364031
Al	1.674324	-0.591382	5.05889
Al	1.908109	1.870568	4.203339
Al	1.2221	1.757653	1.716061
I	1.223324	-4.993349	4.284782
I	-2.373841	0.720755	-1.387408

$Al_9I_3^-$ A

-35.255924 eV

Al	-0.500349	0.084131	0.309718

Al	-0.197493	-0.351688	2.874828
Al	2.144297	-0.136282	-0.16858
Al	2.68883	-0.579927	2.353543
Al	0.76224	-2.216607	0.774779
Al	1.189534	-2.642456	3.26482
Al	1.646549	-0.582444	4.895869
Al	1.728849	1.917693	4.16911
Al	1.237662	1.591444	1.66088
I	1.202673	-4.966284	4.384769
I	-2.402505	0.747113	-1.302341
I	3.434508	0.252394	-2.372413

$Al_9I_3^-$ B

-35.2299

Al	-0.52269	0.118586	0.52348
Al	-0.056218	-0.138796	3.024447
Al	2.232353	-0.124011	-0.006966
Al	2.677425	-0.380966	2.497027
Al	0.802555	-2.173365	0.970672
Al	1.244455	-2.500612	3.432734
Al	1.739404	-0.380494	5.016841
Al	1.784426	2.072108	4.113038
Al	1.27653	1.824225	1.611143
I	-2.148251	0.664399	-1.396004
I	3.123752	0.197156	-2.399098
I	2.150719	-1.096045	7.473117

$Al_9I_3^-$ C

-35.19057266

Al	-0.512815	0.496655	0.238138
Al	-0.260668	-0.390825	2.693538
Al	2.168624	0.40918	-0.190849
Al	2.645263	-0.48435	2.230274
Al	0.852566	-1.789716	0.609971
Al	1.218114	-2.621084	3.107405
Al	1.616296	-1.280163	5.318399
Al	1.507566	1.039999	4.153275

Al	1.154151	1.87893	1.782825
I	0.551101	-3.920174	-0.830464
I	-2.39538	1.250764	-1.35395
I	3.497481	1.062375	-2.305252

Al_{10}^{-}

-28.23151851 eV

Al	-4.324348	-0.699663	-0.181288
Al	-2.225862	0.9936	-0.327741
Al	-1.90594	-1.753286	-0.364252
Al	-0.487977	-0.088363	-2.082585
Al	0.077282	-1.268835	-4.339371
Al	-2.068229	2.432596	-2.519164
Al	-1.422976	0.945152	-4.535824
Al	-1.56819	-2.762549	-2.701381
Al	-2.513288	-1.590831	-4.962048
Al	-3.107083	-0.35466	-2.720915

$Al_{10}I^{-}$

-31.94823579 eV

Al	-1.536458	-0.371474	1.321897
Al	0.661037	1.005696	2.527224
Al	-1.776904	-0.684645	-1.250419
Al	0.034928	3.683904	-1.055128
Al	0.457169	1.01932	0.01107
Al	-2.048801	2.122529	2.634664
Al	-2.059196	2.049526	0.111293
Al	-0.868579	1.592925	-2.239781
Al	0.044398	3.453903	1.533852
Al	-1.256616	0.062835	3.950219
I	-1.639821	-1.008353	6.27049

$Al_{10}I_2^{-}$

-35.05796631 eV

Al	-2.793995	-0.199993	1.420869
Al	0.693767	1.720498	2.718788
Al	-2.114432	-0.177513	-1.174761
Al	1.089289	2.759052	0.186517

Al	-0.053803	0.122677	0.756308
Al	-2.021261	1.922298	3.034672
Al	-1.648662	2.154365	0.40852
Al	-0.062239	1.352064	-1.770188
Al	-0.513928	3.914315	2.030806
Al	-0.845583	-0.39346	3.251208
I	-0.524719	-2.302391	4.955106
I	-0.640933	6.435188	2.563955

Al_{11}^-

-31.75373015 eV

Al	-0.001085	-0.000973	-0.151287
Al	-0.008804	-0.025752	2.75691
Al	2.35897	-0.00971	1.095605
Al	0.899304	2.226764	1.555386
Al	2.51402	0.041035	3.720706
Al	0.87034	2.11861	4.261204
Al	3.325135	2.35172	2.760708
Al	1.871878	4.570311	3.058532
Al	-0.725219	4.017795	2.788473
Al	-1.70761	1.62182	1.104552
Al	-1.7648	1.792179	3.726962

$Al_{11}I^-$

-34.94716973 eV

Al	0.022676	0.137375	-0.112935
Al	0.094672	0.016862	2.668206
Al	2.434394	0.218843	0.98402
Al	0.827126	2.389335	1.373126
Al	2.657795	0.017305	3.601994
Al	0.959999	2.079833	4.154061
Al	3.314631	2.315244	2.493108
Al	1.83505	4.455817	2.996535
Al	-0.774386	3.969661	2.89934
Al	-1.768003	1.601172	1.193755
Al	-1.802874	1.694755	3.8709
I	3.572373	-1.645629	5.386247

$Al_{11}I_2^-$

-38.25094715 eV

Al	-0.053335	0.06333	-0.01143
I	0.03967	-0.048897	2.556199
Al	1.759973	0.007497	-1.867941
Al	-0.563867	-1.693574	-1.853538
Al	2.216097	2.020127	-3.571307
Al	-2.631566	-1.531171	-3.544342
Al	-2.713193	1.035561	-4.269814
Al	-2.239142	3.023868	-2.682143
I	-3.676546	4.988496	-1.841188
Al	-0.211537	2.867866	-4.283286
Al	0.129367	2.232905	-1.596256
Al	-2.190615	0.533202	-1.583226
Al	-0.241726	0.290311	-3.598263

Al_{12}^-

-35.08145187 eV

Al	0.112941	0.156524	0.191695
Al	-0.532376	-0.188135	2.810039
Al	2.755375	-0.151267	0.772619
Al	-0.724149	2.338909	-1.175032
Al	-2.311716	-0.019944	-1.222987
Al	-0.056638	-0.04805	-2.610003
Al	1.905516	1.422474	-1.30719
Al	-2.093532	-1.465887	1.028957
Al	-0.59262	-2.222362	-0.930689
Al	0.892664	-2.116917	1.412316
Al	-2.018745	1.424724	1.141265
Al	1.84729	-1.48635	-1.380678

$Al_{12}I^-$

-38.32734073 eV

Al	-0.042946	-0.019793	-0.064024
I	0.013098	0.019197	2.534864

Al	1.706478	-0.031871	-2.018481
Al	2.411306	-0.128468	-4.665269
Al	1.524026	-2.348029	-3.745467
Al	-0.016173	-2.083718	-1.66621
Al	-0.549835	1.917115	-1.869055
Al	0.356197	1.207695	-5.978989
Al	-0.344577	-0.037058	-3.715125
Al	-2.217365	-0.355465	-1.845775
Al	0.070216	-1.399726	-5.930259
Al	1.411413	2.162696	-3.665816
Al	-1.538453	-2.454093	-3.932927

$Al_{12}I_2^-$

-41.42478679 eV

Al	0.113335	0.14317	-0.008034
I	-0.12106	0.026393	2.574733
Al	2.641666	-0.043731	-0.847539
Al	-1.573655	-0.875595	-1.717981
Al	0.860213	-2.073282	-1.581253
Al	3.409262	-2.242879	-2.026232
I	5.435281	-3.812839	-1.662497
Al	1.947348	-1.783041	-4.219427
Al	-1.627464	0.667522	-4.148404
Al	0.818442	0.300549	-3.063151
Al	3.48941	0.345374	-3.403582
Al	-1.042781	1.805133	-1.81629
Al	-0.694928	-1.862545	-4.007945
Al	1.554431	2.337472	-1.393098

Al_{13}^-

-40.24430458 eV

Al	-0.004978	0.00238	-0.006603
Al	-0.003058	-0.001502	2.673772
Al	2.394056	-0.005191	1.187919
Al	1.937105	1.403139	-1.209735
Al	1.926917	-1.413418	-1.209661
Al	0.746278	2.277715	1.193608

Al	0.731834	-2.279927	1.189884
Al	-1.948448	-1.399804	1.193506
Al	-1.936216	1.419423	1.195435
Al	-0.757383	-2.275148	-1.202257
Al	-2.404402	0.01466	-1.200515
Al	-0.735731	2.286184	-1.203398
Al	-0.012578	0.0005	-2.686678

$Al_{13}I^-$

-42.8167255 eV

Al	-12.052437	-11.893992	-10.022631
Al	-13.401877	-10.597949	-7.933144
Al	-10.779011	-11.127795	-7.784726
Al	-9.400468	-10.919684	-10.047286
Al	-12.173229	-11.331557	-5.497447
Al	-11.538612	-9.165223	-9.457412
Al	-9.122734	-9.019307	-7.980296
Al	-9.954962	-13.434248	-8.891405
Al	-10.028629	-13.08149	-6.111316
Al	-11.59354	-8.827008	-6.674705
Al	-12.430403	-13.229408	-7.589789
Al	-9.510093	-10.361412	-5.549
Al	-8.147743	-11.656807	-7.624121
I	-13.53535	-11.551852	-3.235043

$Al_{13}I_2^-$

-46.25076335 eV

Al	-12.061778	-11.893599	-10.008913
Al	-13.412419	-10.598543	-7.938051
Al	-10.785113	-11.124577	-7.773116
Al	-9.431039	-10.9063	-10.035
Al	-12.165803	-11.326521	-5.526133
Al	-11.551615	-9.157267	-9.430598
Al	-9.133802	-9.013841	-7.95905
Al	-9.952457	-13.427122	-8.87065
Al	-10.02078	-13.088731	-6.105619
Al	-11.594539	-8.817443	-6.655668
Al	-12.4251	-13.240039	-7.591887
Al	-9.506761	-10.367707	-5.538683
Al	-8.154057	-11.646719	-7.618022
I	-8.064917	-10.696712	-12.24831

I	-13.50476	-11.554229	-3.29843

$Al_{13}I_3^-$

-48.76030928 eV

Al	-12.05917	-11.899821	-10.024411
Al	-13.395307	-10.588344	-7.964665
Al	-10.792963	-11.120287	-7.765416
Al	-9.442385	-10.899463	-10.021482
Al	-12.14063	-11.35133	-5.507671
Al	-11.541862	-9.136807	-9.440925
Al	-9.15269	-9.010047	-7.942533
Al	-9.981526	-13.426149	-8.867843
Al	-10.046813	-13.096979	-6.090371
Al	-11.595827	-8.813719	-6.649436
Al	-12.434067	-13.240219	-7.588434
Al	-9.526927	-10.355744	-5.512401
Al	-8.162886	-11.66468	-7.548443
I	-8.057153	-10.709319	-12.207953
I	-13.557158	-11.520398	-3.323795
I	-5.578937	-12.147792	-7.519319

Al_{14}^-

-42.51909568 eV

Al	-0.070904	0.131509	-0.081899
Al	0.062406	-0.120814	2.600401
Al	2.259627	0.122139	-1.394291
Al	-0.920929	-2.204728	1.001603
Al	1.156549	-3.983709	1.122324
Al	1.943576	-1.369659	0.942495
Al	0.559907	-2.103193	-1.386267
Al	1.981264	1.368996	1.109497
Al	-2.077198	-1.142788	-1.300625
Al	-2.413559	0.09053	1.210363
Al	0.732413	2.479334	-1.148178
Al	-0.16805	0.281749	-2.734486
Al	-2.053747	1.667342	-1.081866
Al	-0.650614	2.271002	1.35898

$Al_{14}I^-$

-45.93621028 eV

Al	-0.030029	0.107201	-0.018813
Al	-0.014012	0.094469	2.651549
Al	2.309805	0.03728	-1.302718
Al	-0.910238	-2.097103	1.149091
Al	1.2273	-4.010905	0.778159

Al	1.906509	-1.401611	1.140234
Al	0.53478	-2.131997	-1.314532
Al	1.977195	1.386732	1.143499
Al	-2.084154	-1.206097	-1.210181
Al	-2.428992	0.211183	1.197344
Al	0.744943	2.351199	-1.195622
Al	-0.131919	0.160735	-2.70777
Al	-2.043594	1.538736	-1.22156
I	-3.804206	3.236039	-2.273527
Al	-0.654357	2.383649	1.181478

$Al_{14}I_2^-$

-49.08744986 eV

Al	-0.002239	0.005	-0.004472
Al	0.013289	0.000859	2.638099
Al	2.30092	0.005973	-1.297205
Al	-1.01873	-2.160124	1.244868
Al	1.045933	-3.653323	0.667743
I	1.669955	-6.183922	0.800478
Al	2.052812	-1.295652	1.190964
Al	0.568679	-2.144731	-1.470651
Al	1.939076	1.465057	1.116942
Al	-2.064226	-1.216179	-1.215932
Al	-2.428636	0.156716	1.209093
Al	0.797518	2.334454	-1.265561
Al	-0.131278	0.163655	-2.711416
Al	-2.012235	1.538758	-1.17401
I	-3.765033	3.219717	-2.229648
Al	-0.700424	2.2809	1.271119

$Al_{14}I_3^-$

-52.57759223 eV

Al	-0.017902	-0.05134	-0.003857
Al	0.04169	0.009952	2.625986
Al	2.276822	-0.018085	-1.366567
Al	-1.075593	-2.172127	1.251752
Al	1.011233	-3.637562	0.772567
I	1.732568	-6.145928	0.837468
Al	2.053134	-1.291636	1.163434
Al	0.515816	-2.158247	-1.45893
Al	1.975862	1.433622	1.048275
Al	-2.087517	-1.229433	-1.218923
Al	-2.42853	0.199197	1.198081
Al	0.806834	2.301074	-1.308837
Al	-0.172616	0.144175	-2.719653
Al	-2.025434	1.516172	-1.200687

I	-3.831927	3.134739	-2.224633
Al	-0.623229	2.250927	1.173941
I	1.432989	4.6121	-2.400819
